# Theropod Fauna from Southern Australia Indicates High Polar Diversity and Climate-Driven Dinosaur Provinciality

**DOI:** 10.1371/journal.pone.0037122

**Published:** 2012-05-16

**Authors:** Roger B. J. Benson, Thomas H. Rich, Patricia Vickers-Rich, Mike Hall

**Affiliations:** 1 Department of Earth Sciences, University of Cambridge, Cambridge, United Kingdom; 2 Department of Earth Sciences, University College London, London, United Kingdom; 3 School of Geosciences, Monash University, Melbourne, Australia; 4 Palaeontology Department, Museum Victoria, Melbourne, Australia; Raymond M. Alf Museum of Paleontology, United States of America

## Abstract

The Early Cretaceous fauna of Victoria, Australia, provides unique data on the composition of high latitude southern hemisphere dinosaurs. We describe and review theropod dinosaur postcranial remains from the Aptian–Albian Otway and Strzelecki groups, based on at least 37 isolated bones, and more than 90 teeth from the Flat Rocks locality. Several specimens of medium- and large-bodied individuals (estimated up to ∼8.5 metres long) represent allosauroids. Tyrannosauroids are represented by elements indicating medium body sizes (∼3 metres long), likely including the holotype femur of *Timimus hermani*, and a single cervical vertebra represents a juvenile spinosaurid. Single specimens representing medium- and small-bodied theropods may be referrable to Ceratosauria, Ornithomimosauria, a basal coelurosaur, and at least three taxa within Maniraptora. Thus, nine theropod taxa may have been present. Alternatively, four distinct dorsal vertebrae indicate a minimum of four taxa. However, because most taxa are known from single bones, it is likely that small-bodied theropod diversity remains underestimated. The high abundance of allosauroids and basal coelurosaurs (including tyrannosauroids and possibly ornithomimosaurs), and the relative rarity of ceratosaurs, is strikingly dissimilar to penecontemporaneous dinosaur faunas of Africa and South America, which represent an arid, lower-latitude biome. Similarities between dinosaur faunas of Victoria and the northern continents concern the proportional representatation of higher clades, and may result from the prevailing temperate–polar climate of Australia, especially at high latitudes in Victoria, which is similar to the predominant warm–temperate climate of Laurasia, but distinct from the arid climate zone that covered extensive areas of Gondwana. Most dinosaur groups probably attained a near-cosmopolitan distribution in the Jurassic, prior to fragmentation of the Pangaean supercontinent, and some aspects of the hallmark ‘Gondwanan’ fauna of South America and Africa may therefore reflect climate-driven provinciality, not vicariant evolution driven by continental fragmentation. However, vicariance may still be detected at lower phylogenetic levels.

## Introduction

Early Cretaceous (145.5–99.6 million years ago [Mya]; [1]) tetrapod faunas are of significant interest. Not only do they provide snapshots of ancient ecosystems and document the early evolution of key components of the modern biota (e.g., [2]–[5]), they also immediately postdate the Late Jurassic fragmentation of the Pangean supercontinent (e.g., [6]). Thus, the topology of evolutionary branching events among Early Cretaceous tetrapods may constrain our understanding of continental fragmentation, its interaction with the evolutionary history of major taxonomic groups, and its role in determining the geographic distribution of organisms (via cladistic biogeography; e.g., [7]–[9]). Gondwana, a landmass made up of the present day southern continents and India, became separated from Laurasia (the northern continents) during the Jurassic (e.g., [10]). Timing of the subsequent breakup of Gondwana is not confidently understood (reviewed by [9]). Africa may have separated early, 140–120 Mya, followed by separation of South America from East Gondwana (Australia+Antarctica) in the Late Cretaceous, 80 Mya [11]–[12]. Alternatively, the southern continents may have all separated within a short interval ∼80 Mya [9]. Both hypotheses suggest enduring physical contact between South America, Antarctica and Australia. Thus, prior expectation is that Early Cretaceous tetrapod faunas of Australia should demonstrate biogeographic links with those of Antarctica and South America. Evidence for such links may include both the representation of higher taxonomic groups, and the phylogenetic relationships of individual taxa within those groups.

Compared with those of most other continents, the Australian dinosaur fauna is poorly-known [13]. Thus, it has been neglected in most discussions of Cretaceous biogeography (but see [14]–[17]). However, recent interpretations of the Australian dinosaur fossils have been contentious [15]–[22]. Debate focuses on the inference of a ‘Gondwanan’ fauna [23]–[24], sharing higher taxonomic groups with South America, Africa, Madagascar and India (recently synthesized by [15]). Alternatively, Australian dinosaur faunas may include some classically ‘Laurasian’ clades [16]–[18], [20], reflecting near-cosmopolitanism of high-level taxonomic groups in the Early Cretaceous (e.g., [16]–[17], [20]–[21], [25]–[27]). Early Cretaceous cosmopolitanism is congruent with the Jurassic origination of many groups, known directly from stratigraphic occurrences, and inferred from phylogenetic ghost lineages [28]–[31]. Under this model, clear differences in the higher-taxonomic composition of well-known Late Cretaceous Laurasian and Gondwanan dinosaur faunas [24] arose not via continent-level vicariance, but by differential patterns of regional extinction [27], [32]–[33], and local environmental preferences (e.g., in sauropods [34]–[35]). If this is correct, then the signature of North-South vicariance in Early Cretaceous faunas may be detectable only at lower phylogenetic levels; within, but not among, higher clades of Jurassic origin. This global biogeographic model does not dispute the existence of regional faunas with highly-characteristic taxonomic compositions, such as those of Patagonia [14], [23] and North Africa [36]–[40]. It does suggest, however, that continental fragmentation provides only a partial explanation.

The biogeographic debate surrounding high-level taxonomic assignment of Australian dinosaur fossils (to either ‘Gondwanan endemics’ or elements of a more global fauna) is underpinned by the disputed identifications of many specimens. Debate centres around specimens from the Early Cretaceous (Aptian–Albian) of Victoria, most of which are discovered as isolated elements. For example, the holotypic ulna of *Serendipaceratops arthucclarkei*, and other material, was described as a *Protoceratops*-like basal ceratopsian by Rich & Vickers-Rich [18], [41]. Ceratopsians are only otherwise known from Laurasia (e.g., [42]), and both Australian specimens were recently considered indeterminate within either Genasauria (*S. arthucclarkei*) or Ornithischia [15], although a counter-argument defending the original assignment is currently in preparation (T. H. Rich and colleagues, unpublished data). Most disputed identifications concern theropod dinosaur remains. For example, several Australian theropod specimens have been referred to clades only otherwise known from Laurasia: *Timimus hermani*, based on holotypic and referred femora, has been designated as an ornithomimosaur [41]; NMV P186046, a pair of pubes, may represent a tyrannosauroid [16], [20]; and a surangular (NMV 186386) and dorsal vertebra (NMV P186302) were identified as oviraptorosaurian by Currie et al. [43]. However, the identities of all these specimens have been contested: *T. hermani* was recently reassigned as an unenlagiine dromaeosaurid ([15]: ‘Dromaeosauridae? Indet. cf. Unenlagiinae’); Herne et al. [22] suggested that NMV P186046 might represent an allosauroid; and Agnolin et al. [15] considered the Australian ‘oviraptorosaur’ bones to be indeterminate (NMV P186386) or to belong to a dromaeosaurid (NMV P186302). All these reassignments refer to clades found elsewhere in Gondwana during the Cretaceous, although only Unenlagiinae is restricted exclusively to Gondwana [21], [25]–[26], [44].

Only one Australian theropod is represented by an associated partial specimen, the allosauroid *Australovenator* from the Albian Winton Formation of Queensland (>10° of latitude North of the Victorian dinosaur localities) [45]. *Australovenator* represents Megaraptora, a clade that is becoming well-known from the Cretaceous of South America, but is also represented by *Fukuiraptor*, from Japan [21]. A Victorian ulna (NMV P186076) is closely similar to those of megaraptorans such as *Australovenator* and *Megaraptor* [19], [45] and may indicate that dispersal between South America and Australia remained frequent at the end of the Early Cretaceous [19].

We present a comprehensive survey of theropod postcrania from the Early Cretaceous of Victoria, Australia, and report an abundant collection of teeth from the Flat Rocks Site (38°39′40±2″S, 145°40′52±3″E). Non-dental cranial elements are rare, and only a few have been recovered (the surangular described by [43], and an unidentified frontal), and these are not discussed here because we have little to add in terms of identification. One aim of the present study is to review the disputed affinities of the specimens mentioned above, accompanied by comprehensive figures and detailed comparisons. In addition, many specimens are described here for the first time, and this new data is highly significant for understanding the global biogeographic context of the Australian dinosaur fauna. Fragmentary Early Cretaceous dinosaur remains, including rare theropod elements, are also known from South Australia [46]–[49] and New South Wales (e.g., [46], [50]–[51]), and more complete dinosaur remains from Queensland (e.g., [52]–[54]), including the holotypic partial skeleton of the allosauroid theropod *Australovenator wintonensis* [45]. We provide only a few comments on this additional material. Reviews of Australian dinosaur discoveries of all ages were provided by Rich & Vickers-Rich [55]–[56] and Weishampel et al. [13].

### Institutional abbreviations

AMNH, American Museum of Natural History, New York, USA; IGM, Mongolian Academy of Sciences, Ulaan Baatar, Mongolia; IVPP, Institute of Vertebrate Paleontology and Paleoanthropology, Beijing, China; MACN, Museo Nacional, Buenos Aires, Argentina; MNN, Musée National du Niger, Niamey, Niger; MCNA, Museo de Ciencas Naturales y Anthropológicas (J. C. Moyano) de Mendoza, Mendoza, Argentina; MUCP, Museo de Geología y Paleontología, Universidad Nacional del Comahue, Neuquén, Argentina; NMV, Museum Victoria, Melbourne, Australia; ROM, Royal Ontario Museum, Toronto, Canada; UMNH, Utah Museum of Natural History, Salt lake City, Utah, USA.

### Victorian dinosaur provenance

**Figure 1:**
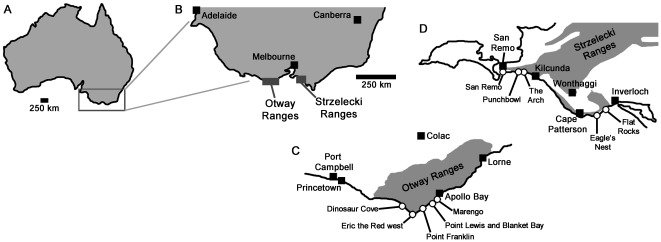
Victorian theropod localities. (A) overview map of Australia, (B) magnification of the southern Victorian (and South Australian) coast showing locations of the Otway and Strzelecki ranges, (C) Otway Ranges locality map, (D) Strzelecki Ranges locality map. Outcrop of the Eumarella Formation (C) and Wonthaggi Formation (D) is indicated with grey shading.

**Figure 2:**
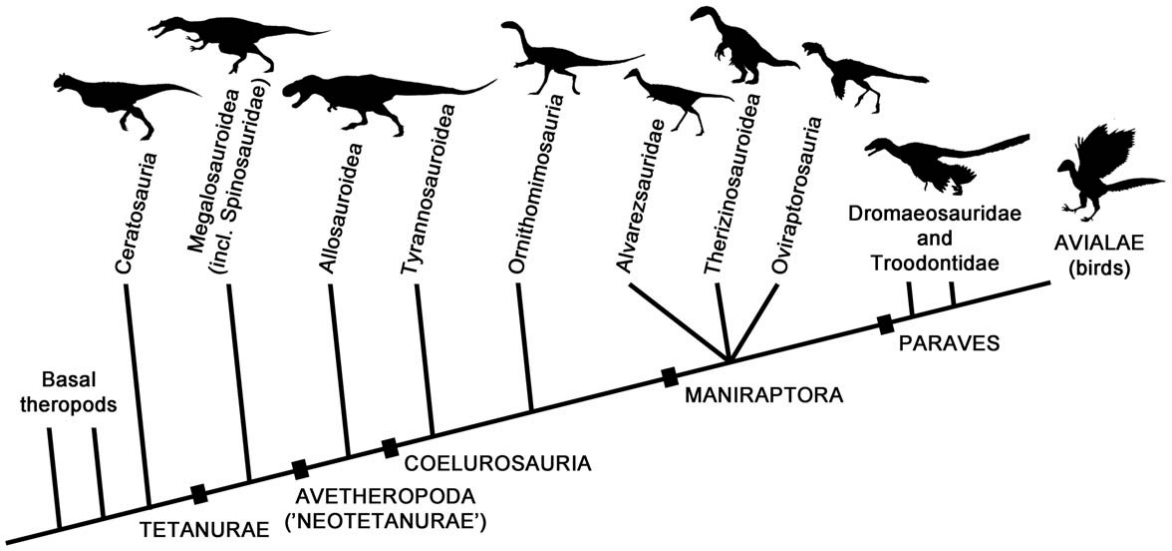
Summary cladogram of theropod relationships.

Victorian dinosaur fossils have been collected extensively from coastal outcrops of two formations (Fig. 1): (1) The Eumeralla Formation (late Aptian–early Albian based on palynology [57]), Otway Group of the Otway Ranges, southwest of Melbourne; and (2) The Wonthaggi Formation (early–middle Aptian [57]), Strzelecki Group of the Strzelecki Ranges, southeast of Melbourne (Vickers-Rich et al. [58]:fig. 1, Rich & Vickers-Rich [59]:fig. 1; [60]:fig. 1, Vickers-Rich & Rich [61]:fig. 2). Both formations were deposited on the northern side of the rift valley formed by North–South extension between Australia and Antarctica 105–125 Mya. The Otway and Strzelecki deposits were originally contiguous, but were later separated by Miocene tectonism. Sediments were derived from a volcanic (andesitic) source, transported primarily during spring thaw floods traveling westwards. Sandstones were laid down by high velocity currents in broad river channels (hundreds of meters wide). Sandstone units reach in excess of 100 meters thickness, comprising a series of channel-fill sands that fine upwards and grade into mudstone-dominated units, commonly containing thin sandstone beds and coal seams. The bases of the sandstone units are marked by scoured surfaces and include mudstone rip-up clasts, abundant plant material, including large logs, and the fossil bones described here. In general, larger bones have been recovered from the eastern, higher-energy part of the depositional system (in the Strzelecki Group).

During the time of deposition, the Victorian coast occupied polar latitudes (75–80 degrees South [62]–[63]). Minimally, this meant at least three months of winter darkness [59], and high levels of seasonality resulting in extreme cold intervals. This is supported by likely cryoturbation structures in the Wonthaggi Formation [64] and oxygen isotope ratios suggesting surface air temperatures close to an annual mean of 0°C [65].

More than 1500 identifiable dinosaur specimens have been recovered from the Aptian–Albian of Victoria, including parts of approximately 250 vertebrae, 500 appendicular elements, 50 ribs, 300 skull elements, 50 euornithopod mandibles or maxilla, 250 euornithopod teeth, >100 theropod teeth, 10 ankylosaur teeth and 4 ankylosaur osteoderms. Theropod, and especially ornithischian, dinosaurs are abundant (ankylosaurs [59]–[60], [66]; ‘hypsilophodontids’ e.g., [59]–[60]). However, sauropods are completely unknown. In both formations, dinosaurs are associated with dipnoans, actinopterygians, testudines, pterosaurs and plesiosaurs (e.g., [59]) as well as mammals [67]–[70]. An enantiornithine bird furcula [71] and the bones and teeth of the temnospondyl *Koolasuchus* are known only from the Wonthaggi Formation [72]–[73]. Crocodiliforms are known from the younger Eumarella Formation [55]. Feathers, a diverse freshwater fish fauna, and invertebrates, including insects, are known from Koonwarra, an inland site in the Strzelecki Group that has not yielded other tetrapod remains [74]–[76].

Most Victorian dinosaur discoveries have resulted from an extended program of field research directed by the Museum Victoria (NMV) and Monash University, assisted by numerous volunteers beginning in 1977 [56], [77]. However, the first discovery, a theropod manual phalanx from the Wonthaggi Formation at Eagle's Nest, was made by mapping geologist William Hamilton Ferguson on 7^th^ May 1903 [56], [78].

Theropod fossils are now known from several localities in the Otway Ranges (Fig. 1C): Eric the Red West (38°51′14″S, 143°32′45″E; also recorded as “Crayfish Bay” and some instances of “Eric the Red” in NMV records); Dinosaur Cove East (38°46′53±1″S, 143°24′14±1″E; sometimes referred to as “Lake Copco” in publications and NMV records); Slippery Rocks (38°46′54±1″S, 143°24′15±1″E); Blanket Bay (38°49′20″S, 143°, 35′13″E); Point Lewis, Lungfish Site (38°49′48″S, 143°35′07″E); Marengo (38°46′46″S, 143°40′00″E); and Point Franklin (38°51′S, 143°33′E).

Theropods are also known from localities in the Strzelecki Ranges (Fig. 1D): Eagle's Nest (38°40.5′S, 145°40.25′E); Flat Rocks Site (38°39′40±2″S, 145°40′52±3″E); The Arch (38°32′54″S, 145°27′29″E); Kilcunda, Black Head (38°33′22″S, 145°28′50″E); 100 metres north of The Caves access (38°39′45″S, 145°40′49″E); Punchbowl (38°32′14″S, 145°24′22″E); and San Remo Back Beach (38°31′54″S, 145°22′13″E).

### Theropod dinosaurs

Non-avian theropod dinosaurs are known from the Late Triassic to the end of the Cretaceous (∼230–65.5 Mya [1]). They were globally-distributed, abundant, and taxonomically diverse, especially in the Jurassic and Cretaceous. All theropods were bipedal, most were carnivorous (e.g., *Velociraptor*), and many attained giant sizes (e.g., *Tyrannosaurus rex*; ∼12 metres long [79]). Theropods included substantial ecological disparity at small and medium body sizes, especially among maniraptoran theropods, which include birds (e.g., [80]–[82]). Because many different theropod clades are discussed in the present work, we provide a schematic phylogeny here (Fig. 2). There is currently little consensus on relationships among basal maniraptoran clades Alvarezsauridae, Therizinosauroidea and Oviraptorosauria (e.g., [31]). Relationships among paravian clades Troodontidae, Dromaeosauridae and Avialae are also uncertain, and it has recently been suggested that ‘unenlagiine dromaeosaurids’ represent a distinct family (Unenlagiidae), more closely-related to Avialae than Dromaeosauridae [83]. However, this is not important for the current study. A detailed account of theropod diversity was provided by Weishampel et al. [84].

## Methods

Most of the material described in this work was collected by an extended program of field research directed by the Museum Victoria (NMV) and Monash University, assisted by numerous volunteers beginning in 1977. All necessary permits were obtained from Parks Victoria for the described field studies.

Because of their preservation as isolated elements, we do not include the elements described here in formal phylogenetic analyses. However, we frequently refer to recent phylogenies and detailed descriptions to interpret the systematic implications of the anatomy described here.

## Results

### Cervical vertebrae

#### NMV P221081, Otway Group, Eric the Red West

**Figure 3:**
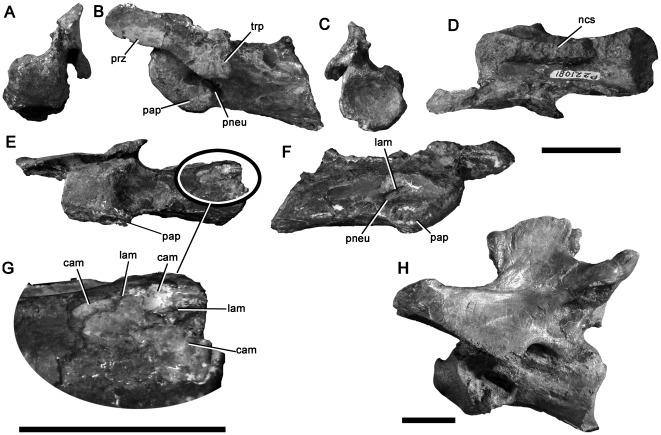
Spinosaurid cervical vertebra NMV P221081 in anterior (A), left lateral (B), posterior (C), dorsal (D), ventral (E) and right lateral (F) views with (G) enlargement showing camerate internal pneumatic structure; (H) cervical vertebra of *Baryonyx* (NHMUK R9951) in left lateral view. Abbreviations: cam, camera [internal chamber within vertebra]; lam, lamina; ncs, neurocentral contact; pap, parapophysis; pneu, pneumatic foramen; prz, prezygapophysis; trp, transverse process. Scale bars equal 20 mm (A–G) and 50 mm (H).

Barrett et al. [17] described a partial cervical vertebra, which they referred to Spinosauridae based on detailed similarity to those of *Baryonyx* [85]. NMV P221081 is not redescribed here in detail, but is figured (Fig. 3), and the evidence for spinosaurid affinities elaborated.

The neurocentral suture of NMV P221081 is unfused. Although the neural arch and centrum are articulated on the left side, the neurocentral contact surface is visible on the right side of the centrum and has a crenulated texture, indicating likely juvenile ontogenetic status. Only the left prezygapophysis is preserved, but this is located dorsolateral to the neural canal, indicating that the prezygapophyses were widely-spaced, as in tetanurans and abelisaurid ceratosaurs, but unlike in more basal theropods in which they are closer to the midline ([86], [87]:character 99). The centrum has a moderately convex anterior surface (opisthocoelous), as in many allosauroids (e.g., [88], [89]) and megalosauroids (e.g., *Marshosaurus*: CMNH 21704), including spinosaurids (*Spinosaurus* [36]; *Suchomimus*: MNN GDF 500), and highly-derived abelisaurids such as *Carnotaurus* (MACN-CH 894). Opisthocoelous cervical centra are also present in alvarezsaurids (e.g., [90]) and *Compsognathus* [87], [91]–[92]. Referral to Alvarezsauridae is unlikely given the absence of a prominent hypapophysis and other derived features of that clade [90]. Referral to the small-bodied taxa Alvarezsauridae and *Compsognathus* is also unlikely because of the relatively large size of NMV P221081. Furthermore, its juvenile status is indicative of even larger adult size.

The centrum of NMV P221081 is more than 2.0 times as long anteroposteriorly (excluding the anterior convexity) as it is high dorsoventrally (Table 1), as in spinosaurids [85] and some coelurosaurs, but unlike in abelisaurids (e.g., [86], [93]), tyrannosauroids [94], and allosauroids, including megaraptorans such as *Aerosteon* (ratio = 1.1, [89]:table 2; [17], [88], [95]; although an ‘elongate’ ratio occurs in *Carcharodontosaurus*, this arises from a reduction in proportional dorsoventral height rather than an increase in anteroposterior length [96]). A single pneumatic foramen is present anteriorly on the lateral surface of the centrum on both sides, as in most tetanurans [97], but unlike in all ceratosaurs, which also possess a second, more posterior opening (e.g., [86], [93]). In Cretaceous allosauroids (Carcharodontosauria), the foramen is often divided by a thick external buttress, such that it forms two distinct openings (e.g., [98]–[100]). This condition is not present in NMV P221081. Instead, the foramen is divided internally, by a fine, subvertical lamina, identical to that in *Baryonyx* [17]. Finally, the ventral surface of the centrum, and parts of the left neural arch, are broken, revealing that NMV P221081 lacks extensive invasion by pneumatic ‘camellae’. Camellae are numerous small internal chambers, characteristic of ceratosaurs and Cretaceous allosauroids (e.g., [21], [99], [101]–[102]). Instead, the centrum of NMV P221081 contains a smaller number of intermediate-sized internal chambers (Fig. 3G: only three–four chambers are present across the entire transverse width of the centrum), as in *Baryonyx* (BMNH R9951), and the neural arch lacks internal chambers altogether.

**Table 1:** Selected measurement (in mm) of theropod vertebrae from the Early Cretaceous of Victoria.

				Centrum			Neural spine	
Specimen	Figure	Locality	Taxon	Length	Height	Width	Height	Max. length
Cervical								
P221081	Fig. 3	Eric the Red West	Spinosauridae	37*	14	16	?	?
Dorsal								
P221187	Fig. 4	Flat Rocks	Neovenatoridae	60	48	36	?	?
P216672	Fig. 5	Flat Rocks	Maniraptora	17	11	9	10	15
P186302	Fig. 6	Dinosaur Cove East	Maniraptora	23	27	30	28	19
P208234	Fig. 7	Dinosaur Cove East	Tetanurae	18	12c−	23c+	?	?
Caudal								
P186168	Fig. 8	Punchbowl	?Ornithomimosauria	50	28 (pos.)	30 (pos.)	?	?
P212840	Fig. 9	Near Kilcunda	Theropoda	?	?	?	?	?
P208096	Fig. 10	Flat Rocks	Neovenatoridae	36	25c+	19c−	?	?
P210090	Fig. 11A–D	Flat Rocks	Theropoda	32	8 (pos.)	9 (pos.)	-	-
P212806		Flat Rocks	Theropoda	29	9	11	-	-
P216642	Fig. 11E–G	Flat Rocks	Theropoda	24	7	10	-	-
P203700		Eagle's Nest	Theropoda	29	14i(pos.)	16 (pos.)	-	-
P185858	Fig. 11H	Dinosaur Cove East	Theropoda	43	8c−	11c+	-	-
P199783	Fig. 11I	Slippery Rocks	Theropoda	44	12c+	9c−	-	-
P229456		Eric the Red West	Theropoda	25	8	11	-	-
P223063		Eric the Red West	Theropoda	23	8	7	-	-

All specimens are housed at the NMV. Abbreviations:

* , measurement excluding convex anterior articular surface, centrum height and width are measured anteriorly; c+, length increased by crushing; c−, length decreased by crushing; (pos.), measurement of posterior articular surface.

### Dorsal vertebrae

#### NMV P221187, Strzelecki Group, Flat Rocks site, Inverloch

**Figure 4:**
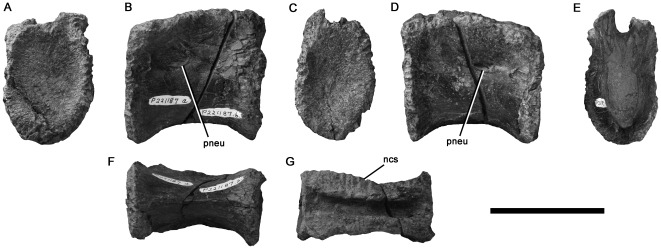
Neovenatorid allosauroid centrum NMV P221187 in anterior (A), left lateral (B), posterior (C), right lateral (D), cross-sectional (E), ventral (F), and dorsal (G) views. (E) shows the a posterior view of a break in the centrum, revealing camellate internal structure. Abbreviations: ncs, neurocentral suture; pneu, pneumatic foramen. Scale bar equals 50 mm.

A centrum that is slightly longer anteroposteriorly than it is high dorsoventrally (Fig. 4) is identified as a dorsal vertebra. The neurocentral contact is visible and has a crenulated texture, indicating lack of fusion and likely juvenile ontogenetic stage despite the large size of the element compared to most other Victorian theropod specimens (Table 1). As preserved, the articular surfaces are approximately 1.25 times as high as they are wide mediolaterally. They are weakly concave and have abraded rims. The lateral surfaces of the centrum converge ventrally to form a low angular longitudinal ventral ridge. Pneumatic foramina are located anterodorsally on the lateral surfaces of the centrum. They are anteroposteriorly elongated with suboval outlines in lateral view and the left foramen is larger than the right. The ventral rims of the foramina project further laterally than the dorsal rims. Thus, they open dorsolaterally rather than strictly laterally.

The vertebra has been sliced into anterior and posterior sections. This reveals an internal structure consisting of a large central chamber, lacking a median septum. Numerous smaller chambers (camellae) are located ventrally and dorsally. These chambers are also visible externally on the abraded portions of the articular rims. The camellate internal structure of NMV P221187 is shared with ceratosaurs, carcharodontosaurian allosauroids (including megaraptorans), tyrannosaurids, oviraptorosaurs and therizinosaurs [101]. All of these clades include taxa with pneumatic middle–posterior dorsal centra like NMV P221187 [103]. However, ceratosaurs possess two pneumatic foramina on the lateral surface of the centrum [93], [97], whereas NMV P221187 has only one, as in tetanuran theropods [97]. Of the remaining clades, NMV P221187 is most similar to carcharodontosaurians and tyrannosaurids in possessing anteroposteriorly elongate, dorsolaterally opening pneumatic foramina located anteriorly on the lateral surface of the centrum (e.g., [79], [89], [99]). We note that the low, angular longitudinal ridge on the ventral surface of NMV P221187 is also present in the sixth and seventh dorsal vertebrae of *Neovenator* [99]:pls 9–10), and the sixth–eighth dorsal vertebrae of *Aerosteon* (MCNA-PV 3137), both neovenatorid carcharodontosaurians. This, combined with the morphology of the pneumatic foramen and internal structure results in detailed evidence, on which basis we refer NMV P221187 to Neovenatoridae indet.

#### NMV P216672, Strzelecki Group, Flat Rocks site, east of ‘The Caves’, Inverloch

**Figure 5:**
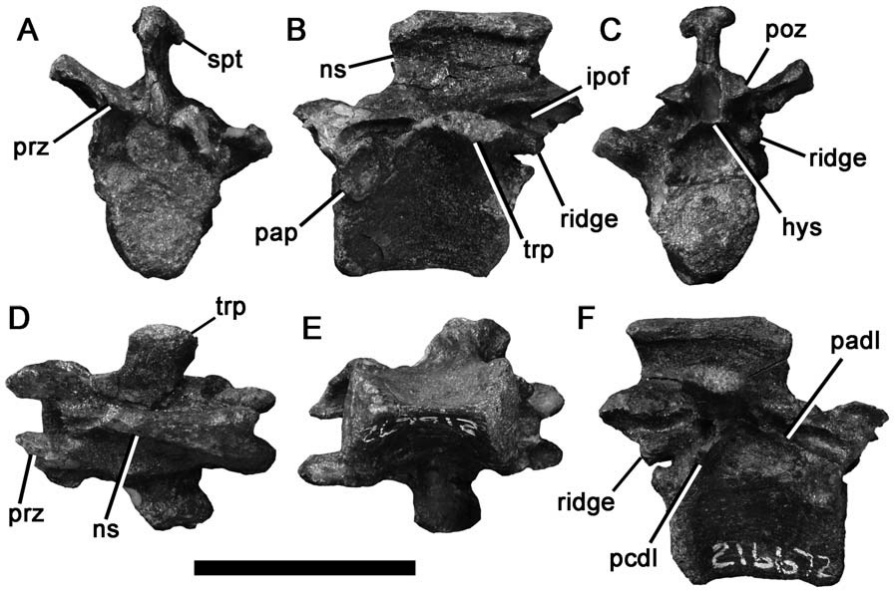
Maniraptoran dorsal vertebra NMV P216672 in anterior (A), left lateral (B), posterior (C), dorsal (D), ventral (E) and right lateral (F) views. Abbreviations: hys, hyposphene; ipof, infrapostzygapophyseal fossa; ns, neural spine; pap, parapophysis; padl, paradiapophyseal lamina; pcdl, posterior centrodiapophyseal lamina; prz, prezygapophysis; spt, spine table; trp, transverse process. Scale bar equals 20 mm.

A small middle or posterior dorsal vertebra (Fig. 5). The neurocentral suture is closed, and is represented by a low rounded ridge, suggesting that NMV P216672 represents an adult individual, despite its small size (Table 1). The centrum lacks a pneumatic foramen, and the parapophysis is located anteroventrally on the lateral surface of the neural arch immediately dorsal to the neurocentral suture. These observations confirm that NMV P216672 is a middle or posterior dorsal vertebra, because anterior dorsal vertebrae are often pneumatic [103] and have a parapophysis that crosses the neurocentral suture.

The articular surfaces of the centrum are slightly taller dorsoventrally than they are wide mediolaterally (Table 1). The anterior surface is weakly concave. The posterior surface appears flat or weakly convex, although this may partly result from abrasion of its ventral rim. The lateral surfaces of the centrum converge ventrally to form a transversely narrow, sheet-like keel that occupies approximately one-third of the centrum height.

The parapophysis is only slightly raised from the lateral surface of the neural arch, unlike those of dromaeosaurids, which are raised laterally on ‘stalks’ (e.g., [104]–[107]). The parapophyseal facet is concave, and has a dorsoventrally long, suboval outline in lateral view. The transverse process extends dorsolaterally. Because of shearing, the left process has been crushed ventrally and the right may have been reoriented dorsally. The transverse process has an approximately rectangular outline in dorsal view, 7 mm wide anteroposteriorly. It bears a robust, laterally oriented buttress on its ventral surface. On the right side, this buttress bifurcates medially to form thin anterior and posterior centrodiapophyseal laminae (terms for neural arch laminae from [108]). On the left side, the anterior centrodiapophyseal lamina is absent and the posterior centrodiapophyseal lamina is a low, robust ridge. The anterior centrodiapophyseal lamina extends anteroventrally at a low angle, thus the neural arch fossae are dorsoventrally low relative to their anteroposterior lengths. This is similar to the condition in small-bodied representatives of several maniraptoran clades, including the unenlagiine dromaeosaurids *Buitreraptor* [25] and *Rahonavis* [109], the troodontid *Saurornithoides* [110] and the therizinosaur *Falcarius* [111]. The neural arch laminae define shallow infraprezygapophyseal, infradiapophyseal, and infrapostzygapophyseal fossae on the right side. These fossae have been cleared of matrix and do not contain foramina. The infrapostzygapophyseal fossa is bisected by a robust, posteroventrally-oriented ridge. The posteroventral portion of this ridge is thick and projects past the posterior opening of the neural canal (Fig. 5). This feature is similar to a ‘bump’ within the infrapostzygapophyseal fossa of posterior dorsal vertebrae in the alvarezsaurid *Patagonykus* ([112]:fig. 5). However, the ridge of NMV P216672 differs from the ‘bump’ in *Patagonykus* because it is separated from the centrum in lateral profile by a distinct notch (Fig. 5B, F).

The neural spine is subequal to the centrum in height and has a low ratio of dorsoventral height to anteroposterior length (ratio = 0.67). Similar, dorsoventrally low, neural spine proportions are present in the therizinosaur *Falcarius* [111], whereas in most other maniraptorans the spine is taller relative to its anteroposterior length (*Mononykus* [113]; *Buitreraptor* [25]; *Microraptor* [114]; *Velociraptor*, [106]). However, the dorsal neural spines of the basal representatives of many maniraptoran groups are not preserved (e.g., *Caudipteryx* [115]–[116]; *Patagonykus* [112]), inhibiting broader comparisons with NMV P216672. The neural spine of NMV P216672 is transversely thin (1 mm anteriorly; 2 mm posteriorly) and sheet-like for most of its dorsoventral height. However, the apex is abruptly expanded, especially posteriorly (mediolateral width = 5 mm) to form a ‘spine table’, which bifurcates posteriorly so it is ‘V’-shaped in dorsal view. The spine table has been considered to be a synapomorphy of Deinonychosauria (Troodontidae+Dromaeosauridae; e.g., [117]:character 108), although Turner et al. ([44]:character 108) found that it was derived independently in dromaeosaurine dromaeosaurids and troodontids. It is also present in alvarezsaurids (*Mononykus* [113], [44]) and the basal therizinosaur *Falcarius* [111], and thus does not help to positively establish the affinities of NMV P216672. However, the spine table is absent in all compsognathids (e.g., [92], [118]).

The prezygapophyses are widely spaced dorsolateral to the neural canal. They extend anteriorly and have dorsomedially facing facets. The postzygapophyses of NMV P216672 have ventromedially facing facets. A hyposphene is formed from thin, sagitally-oriented sheets of bone that extend ventrally from the ventromedial surfaces of the postzygapophyses. These sheets are widely spaced mediolaterally (1.5 mm), divided by a deep midline cleft for most of their height, and only connected ventrally by a horizontal sheet of bone. The presence of this cleft was considered to be a paravian synapomorphy by Rauhut ([87]:character 104), but is also present in some oviraptorosaurs (*Chirostenotes* [119]; confirmed by examination of ROM 43250) and the alvarezsaurid *Patagonykus* ([112]; other alvarezsaurid dorsal vertebrae lack a hyposphene [113], [120]). The hyposhene sheets contact along the midine and are often less widely-spaced and in other theropod clades, including some oviraptorosaurs (*Avimimus* [121]), therizinosaurs [111], ornithomimosaurs [122], tyrannosauroids [79], and other basal coelurosaurs [123], and the condition in compsognathids is unknown.

NMV P216672 is identified as the posterior dorsal vertebra of a maniraptoran, most likely representing an oviraptorosaur, alvarezsaurid or non-dromaeosaurid paravian due to the widely-spaced hyposphene sheets. It has a prominent spine table, as in many maniraptorans [111], [113], [117], and lacks ‘stalked’ parapophyses, a unique, unreversed synapomorphy of dromaeosaurids [106]–[107]. It possesses autapomorphic, posteroventrally oriented ridges within the infrapostzygapophyseal fossae that terminate posteriorly in a ‘bump’ similar to that of the alvarezsaurid *Patagonykus*. However, these structures are not identical. Indeed the condition in NMV P216672, whereby the ‘bump’ does not contact the centrum, is unique and therefore does not allow NMV P216672 to be confidently assigned to Alvarezsauridae. Despite the presence of these autapomorphic ridges in NMV P216672, we refrain from naming a new taxon on the basis of such incomplete material.

#### NMV P186302, Otway Group, Dinosaur Cove East site, Dinosaur Cove

**Figure 6:**
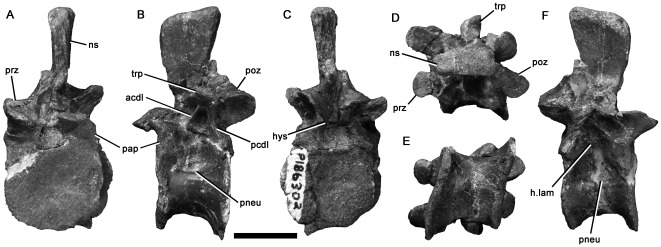
Maniraptoran dorsal vertebra NMV P186302 in anterior (A), left lateral (B), posterior (C), dorsal (D), ventral (E), and right lateral (F) views. Abbreviations: acdl, anterior centrodiapophyseal lamina; h.lam, horizontal lamina; hys, hyposphene; ns, neural spine; pap, parapophysis; pcdl, posterior centrodiapophyseal lamina; pneu, pneumatic foramen; poz, postzygapophysis; prz, prezygapophysis; trp, transverse process. Scale bar equals 20 mm.

A dorsal vertebra was described by Currie et al. [43] as an anterior middle dorsal vertebra (approximately the fifth) of an oviraptorosaur. This specimen is figured here (Fig. 6) but not redescribed in detail.

The original identification as an oviraptorosaur [43] was based on the presence of a relatively low neural spine (Table 1; slightly taller dorsoventrally than the centrum) and the presence of a low ridge on the lateral surface connecting the posterior neural arch peduncle to the parapophyses (Fig. 6: ‘h.lam’), thus forming the ventral margin of the infradiapophyseal fossa [43]. This ridge was reported as present in *Oviraptor*, but absent in dromaeosaurids [43]. Agnolin et al. [15] suggested that these observations indicated, dromaeosaurid affinities instead because (i) the neural spine of NMV P186302 makes up more than 70% of the neural arch height, which they considered comparable to the proportion in dromaeosaurids but taller than in oviraptorosaurs, and (ii) they misreported Currie et al. [43] as stating that the presence of a prominent paradiapophyseal lamina (Fig. 6: ‘padl’) indicated oviraptorosaur affinities, and observed that the paradiapophyseal lamina was more prominent in dromaeosaurids and NMV P186302 than in oviraptorosaurs. Agnolin et al. [15] additionally noted that the pneumatic foramen (‘pleurocoel’) of NMV P186302 was located within a depression, as in some dromaeosaurids, but unlike oviraptorosaurs. These observations are suggestive, but do not provide compelling evidence for dromaeosaurid affinities. We note that the stated differences in neural spine height [15] are minor compared to the level of variation seen within both clades, and that the paradiapophyseal lamina of some oviraptorosaurs (e.g., *Microvenator* [124]) seems equal to that of NMV P186302.

NMV P186302 possesses a single paravian synapomorphy, the presence of broadly separated hyposphene sheets that are connected ventrally by a horizontal lamina (Fig. 6; [87]:character 104). However, this was also derived independently in alvarezsaurids [112] and some oviraptorosaurs [119]. Furthermore, referral to the paravian clade Dromaeosauridae is unlikely as NMV P186302 lacks ‘stalked’ parapophyses (*contra* Agnolin et al. [15]), a dromaeosaurid synapomorphy [105] in which the parapophyses extend laterally approximately as far as the diameter of the parapophyseal facet (e.g., [104], [106]–[107], [114]. Although the parapophyses of NMV P186302 are slightly abraded, their articular surfaces are well-preserved, and it is clear that the absence of the ‘stalked’ condition is not artefactual.

Currie et al. ([43]:fig. 3) provided computed tomographs showing large internal chambers within the centrum and neural arch, open to the exterior of the bone via the pneumatic foramina. This appears somewhat like the simple, ‘camerate’ structure [101]. However, numerous smaller internal chambers in the neural spine, transverse processes, and peripheral parts of the centrum of NMV P186302 represent complex, ‘camellate’ [101] pneumatic chambers [43]. In most camellate taxa, camellae occupy most of the centrum and camerae are thus absent, unlike in NMV P186302. This occurs in at least some oviraptorosaurs ([101]; *contra* [15], [43]) and most other coelurosaurs [101]. However, the condition of troodontid pneumatic vertebrae is unknown, and ‘simple’ (camerate) pneumatic vertebrae, possibly similar to NMV P186302, may be present in the dromaeosaurid *Saurornitholestes* [101].

Based on our current understanding of maniraptoran anatomy, NMV P186302 cannot be confidently excluded from Oviraptorosauria or Troodontidae. However, it lacks the ‘stalked’ parapophyses of Dromaeosauridae, and both articular surfaces are weakly concave, whereas at least one surface is convex in alvarezsaurids [90], [112]. For these reasons, it is considered here as an indeterminate maniraptoran vertebra, possibly representing a troodontid or oviraptorosaur. Numerous clear differences with NMV P216672, such as the proportionally taller neural spine, and presence of a pneumatic foramen in the centrum, indicate the presence of at least two maniraptoran taxa in the Aptian–Albian fauna of Victoria.

#### NMV P208234, Otway Group, Lake Copco site, Dinosaur Cove

**Figure 7:**
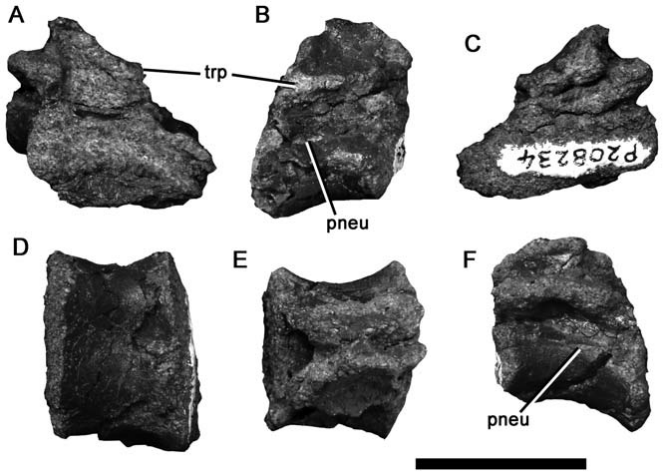
Tetanuran dorsal vertebra NMV P208234 in anterior (A), left lateral (B), posterior (C), ventral (D), dorsal (E), and right lateral (F) views. Abbreviations: pneu, pneumatic foramen; trp, transverse process. Scale bar equals 20 mm.

A small, dorsoventrally crushed dorsal vertebra (Fig. 7; Table 1). The neural arch and the articular surfaces of the centrum are highly abraded. The neurocentral suture is closed, suggesting adult ontogenetic status, despite the small size of NMV P208234. The ventral surface of the centrum is approximately flat, with a weak central depression. The left lateral surface is less crushed than the right and a small, suboval pneumatic foramen is located anteroventrally. A deep recess is present on the ventral surface of the transverse process, extending dorsomedially into the neural arch. This recess contains a small foramen. There is no clear parapophysis on the centrum, but it is clear that if one were present, it did not contact the centrum. This suggests that the parapophysis was present in the abraded anterior end of the lateral neural arch surface. Thus, NMV P208234 represents a middle–posterior dorsal vertebra. Alternatively, it could be the posteriormost dorsal vertebra, which lacks a parapophysis altogether in many theropods. Because many features are abraded, it is difficult to determine the affinities of NMV P208234. However, presence of only a single pneumatic foramen suggests tetanuran affinities [97].

### Caudal vertebrae

#### NMV P186168—Strzelecki Group, 100 metres west of The Punchbowl

**Figure 8:**
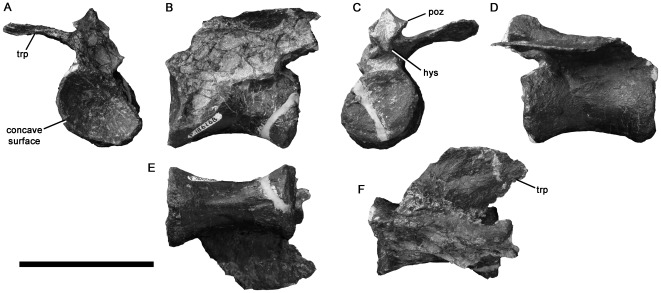
Ornithomimosaur-like caudal vertebra NMV P186168 in anterior (A), left lateral (B), posterior (C), right lateral (D), ventral (E), and dorsal (F) views. Abbreviations: hys, hyposphene; poz, postzygapophysis; trp, transverse process. Scale bar equals 50 mm.

An anteroposteriorly elongate proximal caudal vertebra, with a proplatycoelous centrum (i.e. it has a flat anterior surface and concave posterior surface). The left lateral and dorsal portions of the neural arch are broken and abraded (Fig. 8). The internal structure consists of numerous irregularly-shaped chambers (Fig. 8B). These are not pneumatic, as there are no foramina on the external surfaces that would allow entry for diverticula of the respiratory tract. Although the internal structure of most theropod caudal vertebrae cannot be observed, the same structure seen in NMV P186168 is also present in the ornithomimosaur *Archaeornithomimus* (AMNH specimens). The centrum of NMV P186168 is approximately 1.6 times as long anteroposteriorly as it is high dorsoventrally (Table 1). The anterior surface of the centrum is strongly concave, incompletely preserved and crushed dorsoventrally. The subcircular posterior surface is complete. It is approximately flat, with a shallow concave region dorsally. The ventral rim is thickened, forming an articular area for the chevron that is delimited from the intervertebral articular portion of the surface by a shallow groove. A shallow longitudinal groove is present posteriorly on the ventral surface of the centrum. Other than this, the external (ventral and lateral) surfaces of the centrum are evenly rounded, so the bone would have had a nearly circular cross-section. The neurocentral contact is 40 mm long anteroposteriorly. The neurocentral suture is closed and is only faintly visible in places as a weak crest.

The right transverse process is preserved. It is abraded distally and has been crushed slightly ventrally. The transverse process is dorsoventrally thin, anteroposteriorly elongated (only slightly shorter than the neurocentral contact), and thus sheet-like. It emerges dorsolaterally and is backswept posterolaterally. Prezygapophyses are not preserved, but small, ventrolaterally facing postzygapophyses are present. A small, abraded hyposphene is present, a saurischian synapomorphy among dinosaurs (e.g., [33]). The hyposphene indicates that NMV P186168 is a proximal–middle caudal vertebra, because hyposphenes are absent in more distal caudal vertebrae of all theropods.

Many theropods exhibit anteroposteriorly elongate proximal or anterior middle caudal vertebrae similar to NMV P186168, including ornithomimosaurs [125], dromaeosaurids [104], the basal therizinosaur *Falcarius* [111], and abelisauroids [86], [126]. In contrast, oviraptorids [127], most therizinosaurs [128], [129], alvarezsaurids [90], tyrannosauroids [79], [94], [130]–[131], megalosauroids (e.g., [87], [100], [132]), including spinosaurids [85] and allosauroids [88], [95], including megaraptorans [21], [89], [133], have proportionally shortened proximal caudal vertebrae. Almost all of the taxa listed above have anteroposteriorly narrow caudal transverse processes that are less than half as long anteroposteriorly as the centrum. In contrast, the transverse processes of NMV P186168 are sheet-like and anteroposteriorly expanded, similar to those of some ornithomimosaurs [122], [125], [134]. Although some abelisaurids have anteroposteriorly expanded proximal caudal transverse processes (e.g., [93], [135]), this is associated with an spine-like process extending anteriorly from the lateral end of the process. This process is absent in NMV P186168, and the transverse processes of abelisaurids are longer (laterally) and more robust (dorsoventrally) than those of NMV P186168. Most theropods have amphicoelous caudal vertebrae (e.g., [84], [86], [100]), unlike NMV P186168. Alvarezsaurids have procoelous caudal centra with strongly convex posterior surfaces [90], also unlike those of NMV P186168. Osmólska et al. [122] described the proximal caudal centra of *Gallimimus* as ‘weakly procoelous’. In fact, the anterior surface is concave, whereas the posterior surface is flat or very weakly concave, as in NMV P186168 (S. Brusatte, pers. comm. 2011; R.B.J.B. pers. obs. IGM specimens), and this also occurs in *Archaeornithomimus* (e.g. AMNH 21802).

Based on these comparisons, NMV P186168 uniquely shares two features with the proximal caudal vertebrae of some ornithomimosaurs: proplatycoelous centra and sheet-like, anteroposteriorly expanded transverse processes subequal to the anteroposterior length of the neurocentral suture. The distinctive internal structure is also suggestive of ornithomimosaurian affinities. The vertebral proportions particularly resemble those of the ninth caudal vertebra of *Shenzhousaurus* ([125]:fig. 8). NMV P186168 is identified here as Ornithomimosauria indet. However, despite the strikingly high level of similarity, this identification remains tentative because caudal vertebrae may be difficult to identify precisely.

#### NMV P212840, Strzelecki Group, near Kilcunda

**Figure 9:**
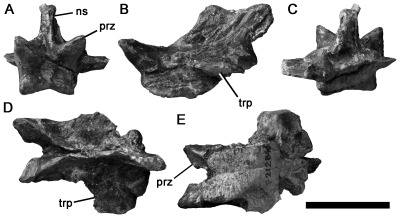
Large theropod caudal neural arch NMV P212840 in anterior (A), left lateral (B), posterior (C), dorsal (D), and ventral (E) views. Abbreviations: ns, neural spine; prz, prezygapophysis; trp, transverse process. Scale bar equals 50 mm.

A large neural arch with broken transverse process and neural spine (Fig. 9). The neurocentral contact measures 47 mm anteroposteriorly as preserved, but was originally slightly longer because the neural arch peduncles are incomplete posteriorly. The neurocentral contact surfaces are covered by a thin layer of matrix, indicating pre-burial disarticulation of the neural arch and centrum. This demonstrates that the neurocentral suture was not fused, suggesting juvenile status, despite the large size of the element.

The neural canal is narrow and is almost completely enclosed by the neural arch. The transverse processes are damaged, but it is clear that they are proportionally short (34 mm) anteroposteriorly compared to those of NMV P186168 (in NMV P212840 they are 0.72 times the preserved anteroposterior length of the neurocentral contact whereas in NMV P186168 these lengths are subequal). The left transverse process is more complete than the right, but has been broken and displaced posteriorly. A robust longitudinal buttress reinforces its ventral surface and bifurcates medially to form low anterior and posterior centrodiapophyseal laminae. These are unlike the prominent centrodiapophyseal laminae of the proximal caudal neural arches of megaraptoran allosauroids, which are separated by deep fossae, often containing pneumatic foramina [21], [89], [133], [136]. However, this observation does not exclude the possibility of megaraptoran affinities because NMV P212840 may represent a slightly more distal caudal vertebra than those preserved in currently-known megaraptoran specimens. The prezygapophyses of NMV P212840 project anterolaterally and have dorsomedially facing facets. A prominent prespinal fossa is located at the base of the neural spine dorsomedial to the prezygapophyses. This extends dorsally as a shallow groove on the anterior surface of the spine for 20 mm. Dorsal to this groove, the anterior surface of the spine is coarsely rugose. The posterodorsally inclined neural spine is transversely narrow (2.5 mm at midlength), but is slightly thickened anteriorly (6 mm) and posteriorly (10 mm). Because the spine is incomplete dorsally, its original height cannot be determined. The ‘anterior spur’ located anterior to the base of the neural spine in the middle caudal vertebrae of many basal tetanurans (e.g., [137]; [87]:character 125) and abelisaurids [86] is absent. However, because of breakage to the neural spine, it is not possible to determine whether a distinct posterodorsal ‘kink’ ([87]:character 123), a serial homologue of the anterior spur, is present (the two structures intergrade continuously along the caudal series [27]). A rugose flange extends from the posterior surface of the spine. The postzygapophyseal facets face ventrolaterally. They are damaged and the left postzygapophysis is displaced laterally. A low, dorsoventrally short hyposphene is present but abraded.

It is difficult to determine the affinities of NMV P212840, because it lacks synapomorphies of any particular clade. Thus it is identified as Theropoda indet.

#### NMV P208096, Strzelecki Group, Flat Rocks Site, Inverloch

**Figure 10:**
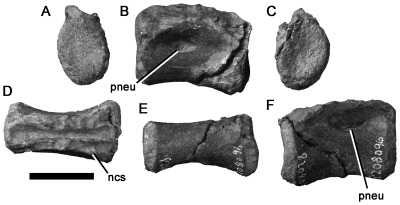
Neovenatorid middle caudal centrum NMV P208096 in anterior (A), left lateral (B), posterior (C), dorsal (D), ventral (E), and right lateral (F) views. Abbreviation: pneu, pneumatic foramen; ncs, neurocentral contact. Scale bar equals 20 mm.

This centrum (Fig. 10) is identified as a middle caudal vertebra because of its relatively elongate proportions (1.5 times as long anteroposteriorly as high dorsoventrally; Table 1). The neurocentral contact is exposed and has a crenulated texture, indicating lack of fusion and likely juvenile ontogenetic stage. The articular surfaces are weakly concave. Due to abrasion, it is difficult to confirm the presence of a chevron facet. The proportions of the articular surfaces have been altered by transverse crushing, but they were probably slightly taller dorsoventrally than wide mediolaterally. The ventral surface of the centrum is evenly rounded, lacking a hypopophysis or ventral ridge or groove. Large, anteroposteriorly long, oval pneumatic foramina (‘pleurocoels’) are located anterior to midlength on the lateral surfaces of the centrum. A break across the centrum allows inspection of the internal structure of NMV P208096. This reveals a complex network of small (∼2–5 mm diameter) internal chambers, corresponding to the ‘camellate’ morphology found in ceratosaurs, carcharodontosaurian allosauroids, tyrannosauroids, therizinosauroids, oviraptorosaurs and many other coelurosaurs [101].

Pneumatic foramina are primitively absent in theropod middle caudal vertebrae [101], [103]. They were acquired independently in megaraptoran allosauroids, oviraptorosaurs, and therizinosauroids. However, in oviraptorosaurs the caudal centra are anteroposteriorly short [127], differing from the elongate proportions of NMV P208096 (Table 1). In therizinosaurs the foramen is small, and located in the neural arch in all but the most proximal caudal vertebrae [128], [138]. Because of these differences with oviraptorosaurs and therizinosauroids, and because of similarity with the middle caudal centra of the megaraptoran allosauroid *Aerosteon*, NMV P208096 is referred to Neovenatoridae indet. Although the basal neovenatorid, *Neovenator*, lacks caudal pleurocoels [99], which are present in megaraptoran neovenatorids like *Aerosteon*, the condition in the phylogenetically intermediate *Chilantaisaurus* is unknown [131], so a precise referral to Megaraptora is not possible on strictly anatomical grounds.

#### Distal caudal vertebrae

**Figure 11:**
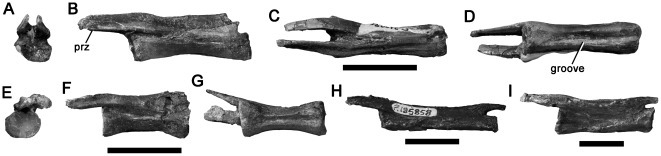
Theropod distal caudal vertebrae NMV P210090 (A–D), P216642 (E–G), P185858 (H) and P199783 (I) in anterior (A, E), left lateral (B, F, H–I), dorsal (C), and ventral (D, G) views. Abbreviation: prz, prezygapophysis. Scale bars equal 20 mm.

Eight distal caudal vertebrae may represent theropod dinosaurs, as they have elongate prezygapophyses, extending further than one-third the length of the centrum anteriorly (Fig. 11) [87], [97]. They are larger than those of small ornithopods such as *Leaeallynasaura* (anteroposterior length of most caudal centra ∼12 mm), which are otherwise represented by abundant material from the Flat Rocks Site and other localities. Although they lack specialised features of certain theropod clades, such as the hyperelongate prezygapophyses of dromaeosaurids (e.g., [87], [104], [107]), the Victorian distal caudal vertebrae do not possess features permitting referral to any particular clade within Theropoda, and are considered as Theropoda indet. herein. Elements of a single general morphology have been recovered from the Strzelecki (Flat Rocks Site: NMV P210090, P212806, P216642; Eagle's Nest: NMV P203700) and Otway (Eric the Red West: NMV P223063, NMV P229456; Slippery Rocks: NMV P199783; Dinosaur Cove East: NMV P185858) groups. The description here is primarily based on NMV P210090, but the other specimens conform to the same morphology (Fig. 11).

The centrum is anteroposteriorly elongate (Table 1). The articular surfaces are weakly concave. The anterior surface is inclined slightly anterodorsally (it is vertical in the other listed specimens). A pronounced longitudinal groove is present on the ventral surface of the centrum. The prezygapophyses are prominent, transversely narrow, and extend 13 mm anterior to the centrum. This represents approximately 0.4 times the centrum length (in another specimen, NMV 216642, the prezygapophyses extend 11 mm anteriorly, 0.45 times the centrum length). The prezygapophyseal facets face ventromedially. The dorsolateral surfaces of the prezygapophyses bear pronounced longitudinal grooves.

### Coracoid

#### NMV P186327, east of The Caves, Inverloch

**Figure 12:**
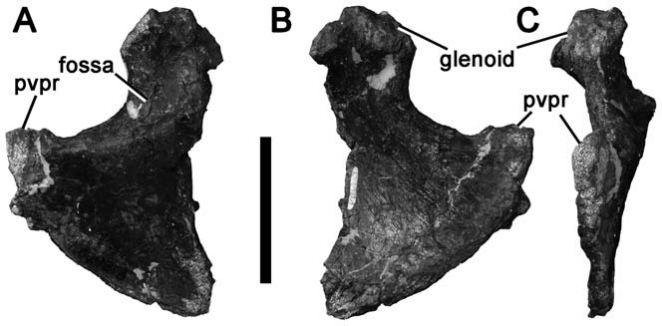
Avetheropodan right coracoid NMV P186327 in lateral (A), medial (B), and ventral (C) views. Abbreviation: pvpr, posteroventral process. Scale bar equals 50 mm.

The ventral portion of a large right coracoid (Fig. 12). This specimen is identified as a theropod dinosaur due to the presence of a fossa on the lateral surface between the glenoid and the posterovental process that is absent in ornithopods (e.g., [139]) and present in some theropods (e.g., *Dilophosaurus* [140]; *Aerosteon* [89]; *Megalosaurus* [100]; some coelurosaurs [44]). Furthermore, although ornithopod coracoids sometimes resemble those of theropods, NMV P186327 is much larger than any ornithopod known from the Victorian fauna. The preserved portion of the bone suggests that the coracoid was longer dorsoventrally than proximodistally, as in non-maniraptoran theropods ([87]:character 137). This contrasts with the proximodistally elongate coracoids of most maniraptorans and birds (e.g., *Microvenator* [124]; *Velociraptor* [106]). The posteroventral process of NMV P186327 tapers to a triangular point, as in coelurosaurs, most allosauroids [88]–[89], [136], [141] and spinosaurids [85]. It is unlike the rounded eminence of most megalosauroids, sinraptorids, and other basal theropods (e.g., [95], [100], [142]), including ceratosaurs (e.g., [93], [143]–[144], [87]:character 136). It is also unlike the extremely elongate posteroventral process of some other coelurosaurs, including ornithomimosaurs and alvarezsauroids [90], [122], [134].

The tapering posteroventral process of NMV P186327 indicates referral to Allosauroidea, Spinosauridae or Coelurosauria. The presence of a fossa between the posteroventral process and glenoid suggests that NMV P186327 does not represent a spinosaurid (spinosaurids lack this fossa: *Baryonyx* [85]; *Suchomimus*, MNN GDF 500). NMV P186327 lacks the subglenoid fossa, an additional fossa that is present in some megaraptorans (*Aerosteon* [89]; *Megaraptor* [136]), but this is absent in some others (*Fukuiraptor* [145]) [21] so its absence in NMV P186327 does not preclude megaraptoran affinities. Although its large size and Early Cretaceous age suggest that NMV P186327 represents an allosauroid, it cannot be distinguished from the coracoids of large-bodied tyrannosauroids on purely morphological ground and is considered here as Avetheropoda indet.

### Ulna

#### NMV P186076, Slippery Rocks Site, Dinosaur Cove

**Figure 13:**
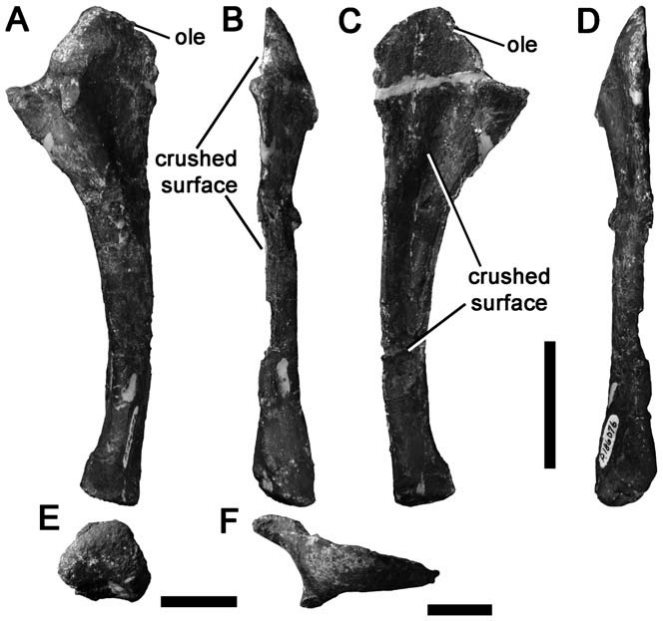
Neovenatorid left ulna NMV P186076 in lateral (A), anterior (B), medial (C), posterior (D), distal (E) and proximal (F) views. Abbreviations: ole, olecranon process. Scale bars equal 50 mm (A–D) and 20 mm (E, F).

A left ulna was figured by Rich & Vickers-Rich ([56]:fig. 8C) and described by Smith et al. [19] as cf. *Megaraptor* (a South American taxon [136], [146]). Although the phylogenetic analysis of Smith et al. [19] placed *Megaraptor* within Megalosauroidea (Spinosauroidea), more comprehensive recent assessments have recovered a derived clade Megaraptora within Allosauroidea [21], [27], [147]. Megaraptorans share several features with megalosauroids due to convergent enlargement of the forelimb and manual ungual phalanges. Despite this potentially confusing homoplasy, all recent authors have agreed that NMV P186076 represents an allosauroid related to *Megaraptor* [15], [21], [27], [45]. NMV P186076 shares numerous detailed similarities with the ulnae of other megaraptorans such as *Australovenator* [45] and *Megaraptor* [136], [146] that have been described several times [19], [21], [45]. Thus, the specimen is not redescribed in detail here, but is figured (Fig. 13). Because the ulnae of basal neovenatorids (the allosauroid family that includes Megaraptora) are not known [99], [131], NMV P186076 cannot be referred specifically to Megaraptora, but is considered as Neovenatoridae indet.

### Manual phalanges

**Figure 14:**
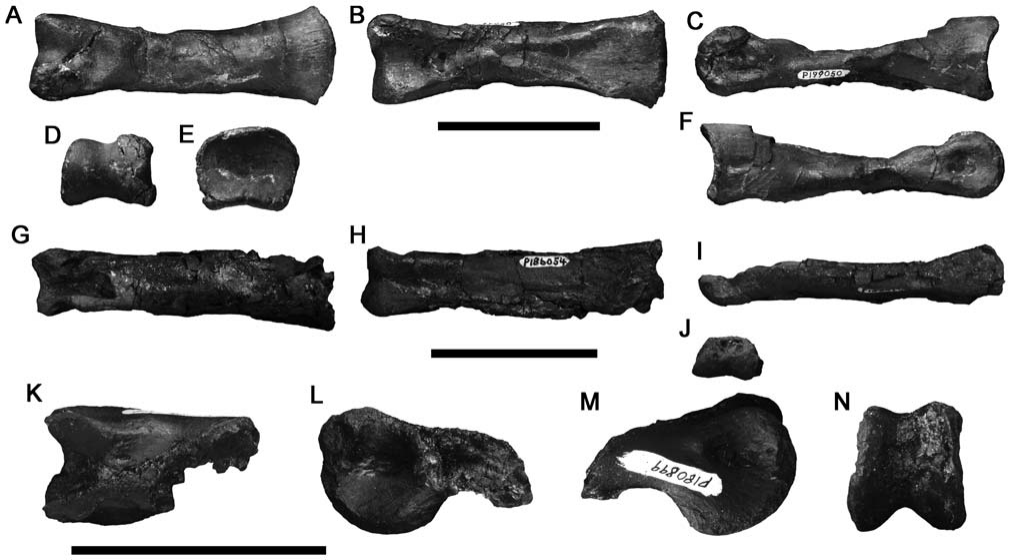
Large theropod manual phalanges NMV P199050 (A–F), P186054 (G–J) and P180899 (K–N) in dorsal (A, G, K), ventral (B, H), left side (C, I, L), distal (D, J, N), proximal (E), and right side (F, M) views. Scale bars equal 50 mm.

Three manual phalanges (Fig. 14) are referred to Theropoda because of the presence of deep, well-defined collateral ligament pits and the presence of an extensor fossa on the dorsal surface proximal to the distal articulation (not preserved in NMV P186054; Fig. 14G–J). We cannot identify the specific identities of these phalanges within the manus because of variability between theropod taxa, and the disarticulated preservation of specimens as isolated bones. However, their generally elongate morphology suggests they represent a theropod with a proportionally long manus, as occurs in megaraptoran allosauroids [21] and many coelurosaurs.

Two of these specimens from the Strzelecki Group (Flat Rocks site: NMV P199050; San Remo: NMV P186054) are similar in size and morphology (Fig. 14). They are proximodistally elongate with a high ratio of proximodistal length to transverse width (e.g., NMV P199050: 4.75; Table 2). Both specimens are crushed dorsoventrally. However, the proximal and distal ends appear uncrushed in NMV P199050, which is better-preserved and is the focus of this description. The ventral surface is flat. The proximal articular surface is divided into subequal medial and lateral concavities by a very weak dorsoventral ridge. The ginglymoid distal articular surface is divided by a shallow, anteroposteriorly oriented sulcus.

**Table 2:** Selected measurements (in mm) of theropod appendicular bones from the Early Cretaceous of Victoria.

Specimen	Figure	Element	Locality	Taxon	Length	Height	Width	Minimum circumference	Distal ap	Distal ml
P186327	Fig. 12	Coracoid	100 N of The Caves	Avetheropoda	93i	94	-	-	-	-
P186076	Fig. 13	Ulna	Slippery Rocks	Neovenatoridae	195	-	-	-	25	21
P199050	Fig. 14A–F	Manual phalanx	Flat Rocks	Theropoda	95	-	-	-	21	28
P180899	Fig. 14K–N	Manual phalanx	Marengo	Theropoda	42	-	-	-	26	23
P186054	Fig. 14G–J	Manual phalanx	San Remo	Theropoda	90	-	-	-	9i	21
P186153	Fig. 15	Manual ungual ?I	West of Punchbowl	Neovenatoridae	103i	-	-	-	-	-
P10058	Fig. 16A–E	Ungual phalanx	200 m W. of Eagle's Nest	Theropoda	52	24i	20	-	-	-
P199085	Fig. 16F–J	Ungual phalanx	Flat Rocks	Theropoda	34i	11	5	-	-	-
P208512	Fig. 16K–O	Manual ungual	Flat Rocks	Theropoda	47	15	8	-	-	-
P186057	Fig. 17	Ilium	Kilcunda	Avetheropoda	98i	30	-	-	-	-
P186046	Fig. 18A–D	Pubes	Slippery Rocks	Tyrannosauroidea	307	-	-	-	139i	21i
P180880	Fig. 18E–I	Pubis	Point Franklin	Coelurosauria	112i	-	-	-	28i	6
P186303	Fig. 19	Femur	Dinosaur Cove East	*Timimus hermani*Tyrannosauroidea	430	-	-	90	48c−	62c+
P186323	Fig. 20A–F	Femur	Dinosaur Cove East	Maniraptora	195	-	-	45	17	23
P180889	Fig. 20G–L	Femur	Lungfish Site	Dromaeosauridae	73i	-	-	-	?	?
P230845	Fig. 20M–O	Femur	Blanket Bay	Coelurosauria	185i	-	-	55	?	?
P150070	Fig. 21A–E	Astragalus	100 m west of Eagle's Nest	Neovenatoridae	64	101i	100	-	-	-
P221202	Fig. 21F–G	Astragalocalcaneum	San Remo	Ceratosauria	31	50	59	-	-	-
P180856	Fig. 22A–F	Metatarsal ?II	Point Franklin	Theropoda	104i	-	-	-	20	13
P209990	Fig. 22G–M	Metatarsal ?IV	Flat Rocks	Theropoda	161i	-	-	-	24	15
P186151	Fig. 23	Pedal phalanx	The Arch	Theropoda	49	?	29	-	?	26

All specimens are housed at the NMV. Abbreviations: I, measurement of incomplete specimen; c+ length increased by crushing; c−, length decreased by crushing.

NMV P180899 (from the Otway Group, 600 metres west of the wooden trig point of navigation beacon at Marengo) is the distal end of a manual phalanx from a larger individual (Table 2). It has a symmetrical, dorsoventrally high, ginglymoid articulation and small, distinct, suboval ligament pits on the medial and lateral surfaces (Fig. 14K–N).

### Manual and undetermined ungual phalanges

#### NMV 186153, first cove west of the Punchbowl, near Kilcunda

**Figure 15:**
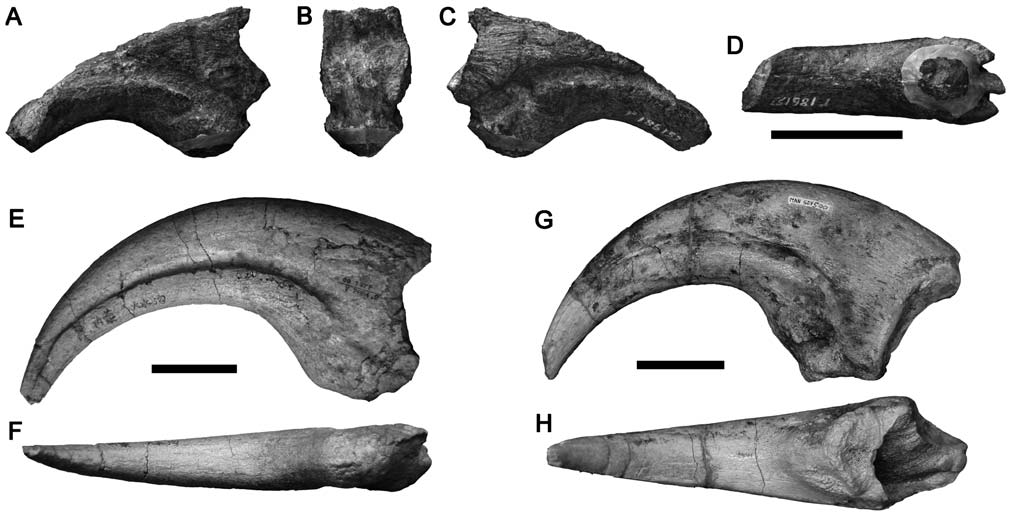
Manual ungual phalanx ?I of a large neovenatorid allosauroid NMV P186153 (A–D) compared to those of the allosauroid *Chilantaisaurus* IVPP V.2884.2 (E–F; modified from [**131**] **) and the spinosaurid megalosauroid** ***Suchomimus*** **MNN GDF 500 (G–H) in left side (A, E, G), proximal (B), right side (C) and ventral (D, F, H) views.** Note that the spinosaurid manual ungual phalanx is transversely broad proximally, unlike the allosauroid and NMV P186153. Scale bars equal 50 mm.

A large manual ungual phalanx missing the distal and proximodorsal portions (Fig. 15A–D). The size of the preserved portion of NMV 186153 is comparable to manual ungual phalanx I of the spinosaurid *Baryonyx* [85] and the allosauroid *Allosaurus* (MACN uncatalogued cast), suggesting that NMV 186153 represents a large-bodied theropod ∼8–9 metres long [85]. The bone is approximately symmetrical. The dorsoventral height of the proximal articular surface cannot be determined precisely due to incomplete preservation dorsally. Comparisons with other large-bodied theropod claws (Fig. 15E–H) [85], [131] suggest that the dorsal half of the bone is missing proximally, and more is missing distally (Fig. 15). The proximal end of NMV 186153 is only slightly broader mediolaterally than is the distalmost preserved portion of the bone, approximately equivalent to midlength prior to breakage (Fig. 15D). This is similar to the condition in neovenatorid allosauroids, such as *Chilantaisaurus*, which have mediolaterally narrow manual ungual phalanges (Fig. 15F) [21], [45], [136], [145]–[146]. It is unlike the condition in more basal allosauroids, such as *Allosaurus* [88], and megalosauroids (e.g., [148]), including spinosaurids like *Baryonyx* and *Suchomimus* (Fig. 15H) [85], which are mediolaterally broad (especially in megalosauroids) and exhibit a substantially distally-tapering outline in ventral view.

A prominent, suboval, mound-like flexor tubercle is present proximally on the ventral surface of NMV 186153. Broad vascular grooves are present on both lateral and medial surfaces. These are deep and well-defined compared to those of many other large-bodied theropods (e.g., [45], [85], [88]), and especially compared to those of large-bodied tyrannosauroids such as *Dryptosaurus* [149] and *Tyrannosaurus* [79]. An independent, Early Cretaceous, origination of large body size in tyrannosauroids is documented by the Chinese taxon *Sinotyrannus* [150]–[152]. However, *Sinotyrannus* has relatively unenlarged and weakly recurved manual ungual phalanges compared to NMV 186153 [150].

The large size of NMV P186153 suggests referral to either Megalosauroidea or Allosauroidea, both theropod clades that produced large-bodied taxa with enlarged forelimbs and giant claws [21], [87], [148]. NMV P186153 is transversely narrow, similar to neovenatorid allosauroids and unlike the proximally broad, distally tapering manual unguals of megalosauroids including spinosaurids. However, it is not as proportionally narrow as the manual ungual phalanges of megaraptoran neovenatorids (e.g., [136], [146]). Thus, it is referred to Neovenatoridae herein, and may not represent a megaraptoran.

#### NMV P10058, 200 metres north west of Eagle's Nest

**Figure 16:**
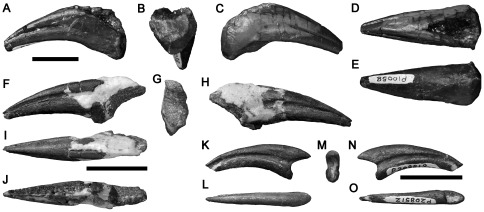
Theropod ungual phalanges NMV P10058 (A–E), P199085 (F–J) and P208512 (K–O) in left side (A, F, K), proximal (B, G, M), right side (C, H, N), dorsal (D, I, L), ventral (E, J, O) views. Scale bars equal 20 mm.

An undetermined ungual phalanx missing the proximodorsal portion (Fig. 16A–E) was reported by Woodward in 1906 [78] as a specimen of *Megalosaurus*. This was the first dinosaur specimen collected in Australia [56]. It has a rounded, subtriangular cross-section, formed by the dorsolateral and dorsomedial surfaces, and a flat ventral surface. In many theropods, the manual ungual phalanges have narrow, convex ventral surfaces, resulting in a suboval, rather than subtriangular, cross-section (e.g., [104], [131]). Thus, NMV P10058 may be a pedal ungual phalanx. However, the triangular cross-section also resembles the manual ungual phalanges of ornithomimosaurs (e.g., [134], [153]) and alvarezsaurids [90]. Thus, its identity (either manual or pedal) remains uncertain. The phalanx is highly asymmetrical, its ventral surface is inclined to one side (it is not certain whether it faces ventromedially or ventrolaterally), and the vascular groove on that side is located futher dorsally than that of the other side. A low, rounded flexor tubercle is present proximally on the ventral surface, distal to the proximal articular surface. Distal displacement of the manual ungual flexor tubercles to approximately one-third of the phalanx length is an ornithomimid synapomorphy ([154]:character 33), but the degree of displacement in NMV P10058 is less than this. Because NMV P10058 lacks synapomorphies of any particular clade, it is considered as Theropoda indet. here.

#### NMV P199085, Flat Rocks Site

NMV P199085, an undetermined ungual phalanx (Fig. 16F–J). It is transversely broader ventrally than it is dorsally, with a flattened ventral surface similar to those of theropod pedal ungual phalanges, or the manual ungual phalanges of ornithomimosaurs and alvarezsaurids. It is only weakly curved. The proximal articular surface bears matrix. It is inclined to face proximoposteriorly, because the dorsal portion projects further proximally than the ventral portion. A low flexor tubercle is present in NMV P199085. It is damaged and so incomplete mediolaterally, but the original anteroposterior height is represented completely or almost completely. Deep vascular grooves extend along the medial and lateral surfaces of the bone. Because it lacks synapomorphies of any particular clade, it is considered Theropoda indet. here.

#### NMV P208512, Flat Rocks Site

NMV P208512 (Fig. 16K–O), a manual ungual phalanx, incomplete proximally and distally. It is transversely narrow for its entire dorsoventral height, and only weakly curved. The proximal articular surface is eroded. The flexor tubercle is not preserved. Deep vascular grooves extend along the medial and lateral surfaces of the bone. NMV P208512 cannot be identified to a particular theropod clade and is considered Theropoda indet. here.

### Ilium

#### NMV P186057, east side of ‘The Arch’, near Kilcunda

**Figure 17:**
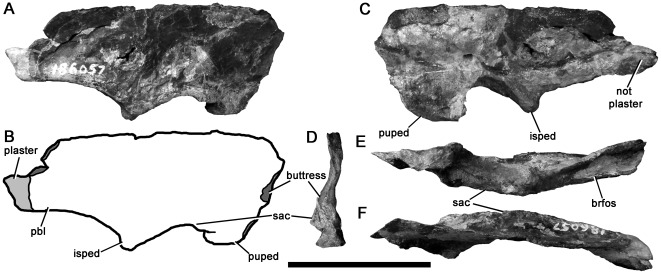
Avetheropod right ilium NMV P186057 in lateral (A–B), medial (C), anterior (D), ventral (E), and dorsal (F) views. In line drawing (B), dark grey tone indicates broken surface, light grey tone indicates restoration. Abbreviations: brfos, brevis fossa; isped, ischial peduncle; pbl, posterior blade; puped, pubic peduncle; sac, supraacetabular crest. Scale bar equals 50 mm.

A partial right ilium missing the anterior and posterodorsal portions of the blade (Fig. 17). This specimen has been crushed slightly mediolaterally. The ilium is long and low, with a ratio of anteroposterior length to dorsoventral height above the acetabulum greater than 3.0. The dorsal margin of the blade is damaged, but seems to be almost complete. The lateral surface of the blade has been fractured and distorted by transverse crushing so it appears undulous. Although it is likely that it was originally smooth, it is difficult to determine if subtle topographic features were present. However, it is clear that a median vertical ridge as seen in tyrannosauroids [130], [155]–[156], many allosauroids and megalosauroids [142] ([100]:character 162), and some ornithomimids and therizinosaurs [157] is absent.

The pubic peduncle is incomplete anteriorly, but is nonetheless substantially larger than the tapering ischial peduncle, and is anteroposteriorly long (21 mm) relative to its mediolateral width (8 mm), resulting in a length∶width ratio of 2.6. This is substantially greater than the ratio in non-avetheropod theropods, and also somewhat greater than in allosauroids such as *Aerosteon* and *Allosaurus* (ratio = 2.0; [89]:table 3; [100]:table 3). This high ratio may indicate coelurosaurian affinities ([87]:character 175). However, the ratio is also high in some juvenile specimens (such as the holotype of *Iliosuchus incognitus* [158]), raising the possibility that NMV P186057 is a juvenile allosauroid ilium. The pubic articular surface of NMV P186057 is only preserved posteriorly. It faces ventrally, similar to carcharodontosaurian allosauroids (e.g., [89]) and many coelurosaurs. A large, anterolaterally facing preacetabular fossa was likely present, but is broken. This is indicated by the preserved base of a mediolaterally thick, anteriorly oriented buttress on the lateral surface anterodorsal to the pubic peduncle that would have formed the dorsal margin of the fossa (Fig. 15A–B). The presence of a preacetabular (‘cuppedicus’) fossa is an avetheropodan synapomorphy ([157], [100]:character 164).

The supracetabular crest of NMV P186057 is low, similar to those of many coelurosaurs [87] and some allosauroids, such as *Aerosteon* [89] and *Giganotosaurus* (MUCPv-Ch 1). Because of transverse crushing, the crest is oriented ventrolaterally. However, it is likely that it was more laterally oriented prior to crushing. A deep brevis fossa is present ventral to the posterior portion of the iliac blade. The medial and lateral walls of this fossa are equal in height, so that in lateral view the medial wall is concealed by the lateral wall.

The condition in NMV P186057, whereby the medial wall of the brevis fossa is concealed in lateral view, is present in megaraptoran allosauroids [89], and in many maniraptorans (e.g., paravians, [87]:text-fig. 39; oviraptorosaurs, [159]:text-fig. 5; *Anchiornis* [160]; *Falcarius* [111]). It is also present in ‘*Iliosuchus*’, which likely represents a juvenile individual of *Megalosaurus* [158]. In many other theropods, including ontogenetically mature *Megalosaurus* specimens [100] and many coelurosaurs, the anterior portion of the medial wall of the brevis fossa often extends further ventrally than the lateral wall, and is thus exposed in lateral view (e.g., [87]:text-fig. 39; *Tyrannosaurus* [79]; *Alioramus* [94]; *Guanlong* [130]; *Xiongguanlong* [161]; *Garudimimus* [162]; *Sinornithomimus* [154]; *Shenzhousaurus* [125]).

NMV P186057 shows tetanuran features such as a proportionally large pubic peduncle, and avetheropodan features such as a preacetabular fossa and anteroposteriorly elongate pubic peduncle. It lacks features seen in many maniraptorans, such as a reduced, medially oriented medial wall of the brevis fossa (e.g., [111], [164], [163]–[166]). Other than this, it lacks diagnostic features that would allow assignment to a specific basal avetheropodan clade, and we consider it as Avetheropoda indet.

### Pubes

#### NMV P186046, Slippery Rocks Site, Dinosaur Cove

**Figure 18:**
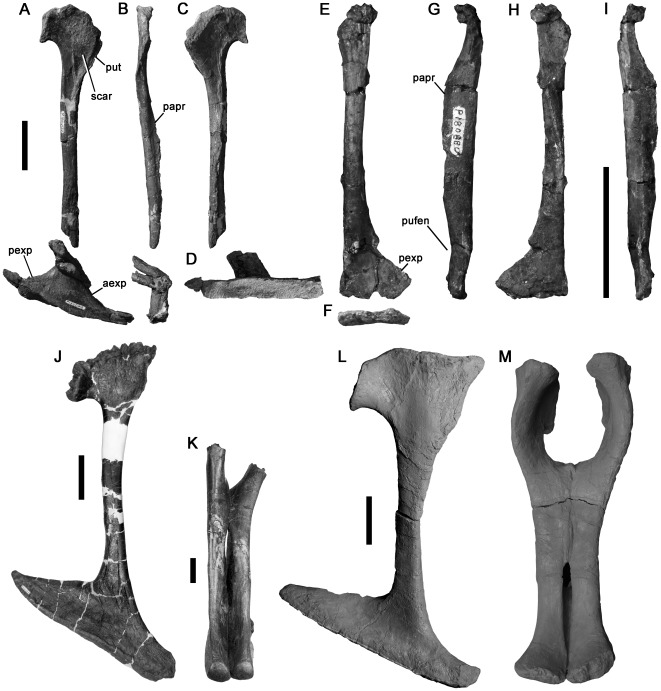
Theropod pubes. Tyrannosauroid right pubis and conjoined distal expansions NMV P186046 (A–D), coelurosaurian left pubis NMV P180880 (E–I), albertosaurine tyrannosauroid right pubis (J), *Tyrannosaurus* conjoined mid-distal pubes (K; modified from Brochu 2002), and casts of pubes of the allosauroid *Aerosteon* (L–M) in lateral (A, E, J, L), anterior (B, G, K, M), medial (C, H), distal (D, F), and posterior (I) views. Abbreviations: aexp, anterior expansion; papr, pubic paron; pexp, posterior expansion; pufen, interpubic fenestra; put, pubic tubercle. Scale bars equal 50 mm (A–I) and 10 mm (J–M).

Articulated pubes, including most of the right pubis, fragments of the left pubis, and the conjoined pubic distal expansions (Fig. 18A–D). NMV P186046 was described as a tyrannosauroid by Benson et al. [16] based on the presence of an anterolaterally curving, flange-like pubic tubercle, a broad, suboval muscle scar on the lateral surface of the pubis posterior to the tubercle, and the presence of a proportionally large distal expansion that is expanded both anteriorly and posteriorly. Both features are present in derived tyrannosauroids, including tyrannosaurids (e.g., [16], [79], [151], [155], [165]). Although the large distal expansion is also present in some allosauroids, the transversely narrow morphology is a unique synapomorphy of Coelurosauria (compare Fig. 18J–K with Fig. 18L–M; [87]:character 185) and precludes referral to Allosauroidea. Furthermore, although many archosaurs, including all theropods, possess a pubic tubercle [157], the morphology of the pubic tubercle of NMV P186046 is unique to tyrannosauroids (Fig. 18J–K) [16], [79], and absent in other theropods, including allosauroids (Fig. 18L–M) (e.g., [88]–[89]). Herne et al. [22] doubted the referral of NMV P186046 to Tyrannosauroidea, arguing that the transversely narrow morphology could result from crushing, and that the pubic tubercle was too poorly-preserved to determine its morphology. However, Benson et al. ([20]:fig. 1) provided additional images, confirming the flange-like morphology of the pubic tubercle. They also calculated that the distal expansion of allosauroids, including megaraptorans, was proportionally three times as broad mediolaterally as those of NMV P186046 and tyrannosauroids. The broad, non-coelurosaurian morphology occurs in juvenile and adult allosauroid specimens alike (several ontogenetic stages of *Allosaurus* pubis from the Cleveland-Lloyd Quarry are accessioned at UMNH), so the narrow morphology of NMV P186046 does not simply reflect ontogeny. Furthermore, there is no evidence for the extreme amount of crushing required to ‘transform’ an allosauroid pubis into a transversely narrow, coelurosaurian pubis like NMV P186046. Finally, breakage to the distal portion of the distal expansion would not give the artefactual appearance of a narrow structure. Thus, we uphold the referral of NMV P186046 to Tyrannosauroidea herein.

#### NMV P180880, Point Franklin shore platform, Cape Otway

A right pubis, preserving the shaft and proximal part of the distal expansion (Fig. 18E–I) is quite small, thus representing an individual substantially smaller than NMV P186046. The shaft is approximately straight in anterior and lateral views. The proximal and distal ends are broken. Despite incomplete preservation, it is clear that the puboischiadic membrane was not ossified (the pubic apron migrates posteroproximally and merges with the pubic shaft; this contrasts with the morphology of taxa with an ossified puboischidic membrane, in which it does not merge with the shaft, but instead becomes more prominent to form the obturator flange). Thus, pubic and obturator foramina were absent, as in most avetheropods ([97], [87]:character 181). A crest-like pubic apron originates as a prominent flange extending from the posteromedial surface of the shaft. It migrates anterodistally to form a medial crest on the anteromedial surface of the shaft. This crest merges with the medial surface of the shaft at two-thirds of the preserved length of the bone. The medial surface of the pubis distal to the pubic apron is recessed laterally, so a proximodistally elongate, slit like interpubic fenestra would have been present, representing a unique synapomorphy of Tetanurae ([21], [87]:character 182). The distal expansion is transversely narrow; despite incomplete preservation of the anteroposterior length, it is more than four times as long anteroposteriorly as it is wide mediolaterally, as in coelurosaurs [87]. The distal expansion is considerably expanded posteriorly, so it measures approximately 4.0 times the minimum anteroposterior shaft diameter. Because it is incompletely preserved, this is a minimum value, and it is possible that NMV P186046 originally possessed a prominent, ‘hook-like’ posterior expansion similar to those of compsognathids and other basal coelurosaurs (e.g., [92], [123]). Unlike in NMV P186046, as preserved, the distal end of NMV P180880 does not expand anteriorly.

NMV P180880 possesses tetanuran, avetheropodan and coelurosaurian synapomorphies described above that allow confident referral to Coelurosauria. Most coelurosaurs possess at least a slight anterior expansion of the distal pubis that is apparently absent in NMV P180880. However, the distal end of NMV P180880 is incomplete, and an anterior expansion may have been located slightly further distally than the preserved portion. Many coelurosaurs have a straight pubic shaft, including basal taxa such as *Compsognathus* [91], basal tyrannosauroids [156] and some ornithomimosaurs [122]. On this basis, NMV P180880 is assigned to Coelurosauria indet. However, it can be excluded from various derived clades that possess a curved, or otherwise distinctive pubic shaft. Oviraptorosaurs and therizinosaurs have an anterodistally curving pubis (e.g., *Enigmosaurus* [129]; *Microvenator* [124]; *Nomingia* [127]; the pubic shaft is sinuous in *Falcarius* [111]). In dromaeosaurids the distal expansion is inclined posterodistally, so the anterior margin of the distal pubis is convex (*Bambiraptor* [166]; *Buitreraptor* [25]), and the distal expansion of troodontids is proximodistally elongate [167]. All are unlike NMV P180880. The pubis is anteriorly convex in the ornithomimosaurs *Garudimimus* [162] and *Shenzhousaurus* [125], and the alvarezsaurid *Patagonykus* [112]. It is anteriorly concave in derived tyrannosauroids ([151]:character 269). And finally, in birds and derived alvaresaurids [90] the pubis is reduced to a splint.

Because of the presence of coelurosaurian symapomorphies and absence of the features of derived coelurosaurian clades, we refer NMV P180880 to Coelurosauria indet. and suggest it represents a relatively basal coelurosaur similar to compsognathids or *Coelurus*.

### Femora

#### NMV P186303, Lake Copco Site, Dinosaur Cove

**Figure 19:**
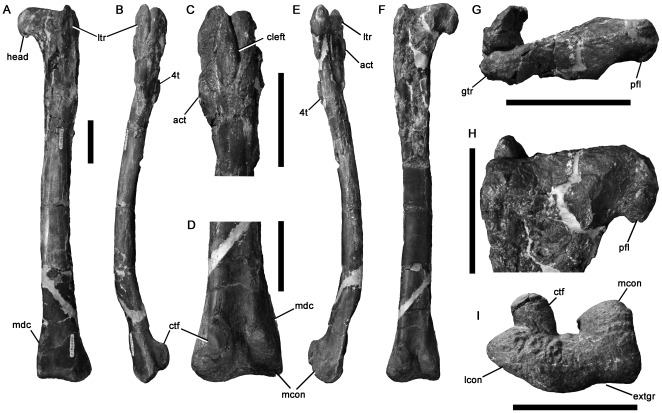
Holotypic left femur of *Timimus hermani* NMV P186303 in anterior (A), lateral (B–C), posterior (D, F), medial (E), proximal (G), posteroproximal (H) and distal (I) views. Abbreviations: 4 t, fourth trochanter; act, accessory trochanter; ctf, crista tibiofibularis; extgr, extensor groove; lcon, lateral condyle; ltr, lesser trochanter; mcon, medial condyle; pfl, posterior flange. Scale bars equal 50 mm.

A left femur was made the holotype of *Timimus hermani* by Rich & Vickers-Rich [41]. The shaft is straight in anterior view and curves posterodistally (Fig. 19). It is extremely long and slender, with a ratio of proximodistal length to minimum mediolateral width of 12.6. This is similar to the ratio in the dromaeosaurid *Unenlagia* (14.7, F. E. Novas, pers. comm. October 2011) [15], [168]–[169], some troodontids (e.g., *Saurornithoides* [170]:∼13.0), noasaurid ceratosaurs (e.g., *Masiakasaurus* [126], [171]:∼12.0) and some ornithomimosaurs (*Struthiomimus* [172]:∼12.5).

The shaft of NMV P186303 is crushed anteroposteriorly, especially the proximal half, which has been slightly reoriented so that its posterior surface faces slightly posterolaterally. The femoral head is oriented strictly medially, and inclined proximally, relative to the posterior surface of the proximal femur. This likely represents its original orientation, because the crushing does not seem so pronounced as to have substantially reoriented the femoral head. The posterior flange of the caput is low and rounded. It does not extend further posteriorly than the lateral portion of the head. As in non-maniraptoran theropods [173] ([87]:character 196), including tyrannosauroids (e.g., [79]:fig. 95I) and ornithomimosaurs (e.g., [154]:fig. 21E), the greater trochanter is anteroposteriorly narrow (15 mm), compared to the widest portion of the femoral head (22 mm). These measurements accurately reflect the morphology prior to crushing; although the proximal part of NMV P186303 is crushed, resulting in reorientation of the dorsal (proximal) surface of the femur to face posterodorsally, there is no indication that the overall width has contracted, or that the greater trochanter was crushed more strongly than the femoral head. In fact, the femoral head seems to have been most strongly affected by crushing.

The lesser trochanter has been crushed slightly anteroposteriorly, but is not substantially deformed. It is tall, extending further proximally than the greater trochanter as in many coelurosaurs [87], but unlike more basal theropods including the megaraptoran *Australovenator* [45]. The lesser trochanter is separated from the greater trochanter by a cleft for at least half its proximodistal length (Fig. 19C), as in most non-paravian coelurosaurs (fusion of the trochanters for most of their length occurs in paravians and some alvarezsaurids [87], [90] and oviraptorosaurs). The accessory trochanter is a transversely thickened region occupying the distal half of the otherwise narrow anteromedial surface of the lesser trochanter. In profile, it forms a low, rounded, proximodistally elongate projection. The morphology of the accessory trochanter and the relatively anteroposteriorly narrow lesser trochanter of NMV P186303 are similar to those of derived tyrannosauroids such as *Xiongguanlong* [161] and tyrannosaurids (e.g., [79], [94]). They are unlike the anteroposteriorly broad, ‘aliform’ lesser trochanter and prominent, triangular accessory trochanter of allosauroids (e.g., [45], [88], [99]), ornithomimosaurs (e.g., [122], [134]), basal tyrannosauroids such as *Guanlong* ([130], [151]:character 287), and other basal coelurosaurs such as *Tanycolagreus* [123]. The fourth trochanter of NMV P186303 is a semioval crest that extends posteriorly from the posteromedial surface of the femoral shaft between 75 and 130 mm from the proximal surface of the head. It is not reduced to a low ridge, and is thus unlike the fourth trochanter of maniraptorans and some ornithomimosaurs ([122], [134], [87]:character 201). The medial surface of the fourth trochanter is rugose.

The distal condyles are crushed anteroposteriorly. As in other theropods, the medial condyle extends further posteriorly than the lateral condyle. However, the crista tibiofibularis, a large tuberosity on the posterior surface of the lateral condyle, extends further posteriorly than the medial condyle. The crista is divided from the posterior portion of the medial condyle by a broad flexor groove. The femoral condyles are separated distally by a broad trough, extending the full anteroposterior length of the distal surface of the femur, an avetheropodan synapomorphy ([21]:character 233). The anterior surface of the distal femur is approximately flat, bearing only a weak extensor groove. Within Tetanurae, the absence of a deep extensor groove is a coelurosaurian synapomorphy ([100]:character 193; the groove is also absent in non-tetanuran theropods and the megalosauroid *Dubreuillosaurus* [174]). This is reversed in derived tyrannosauroids, including tyrannosaurids, in which a deep extensor groove is present (e.g., [79], [151], [161], [175]). The sharp, but low, medial distal crest extends medially from the anteromedial surface of the distal femur. It is approximately 70 mm long proximodistally. Adjacent to the medial distal crest, the medial part of the anterior surface is occupied by a shallow concave region scored by weak longitudinal striations.

*T. hermani* can be referred to Avetheropoda based on the morphology of the proximal surface, lacking an oblique ‘articular groove’ ([100]:character 188), and that of the distal surface, lacking a central depression, but possessing a pronounced anteroposterior trough separating the condyles ([158], [21]:character 233). Within Avetheropoda, it can be confidently referred to Coelurosauria based on the presence of a lesser trochanter that extends proximal to the greater trochanter, and the absence of a deep extensor groove [87], [100]. It further differs from allosauroids, including megaraptorans (e.g., [45], [88]) because the medial distal crest and associated muscle scar are both relatively weak, and the tibiofibular crest projects further posteriorly than the medial condyle.

Agnolin et al. [15] referred *T. hermani* to ‘Dromaeosauridae? indet. cf. Unenlagiinae’ based on the absence of the fourth trochanter and appression of the lesser and greater trochanters. In fact however, the fourth trochanter is prominent compared to those of dromaeosaurids, and the lesser and greater trochanters are well-separated (Fig. 19; although they may have been slightly crushed together). Furthermore, *T. hermani* lacks the anteriorly bowed, proximomedially curving femoral shaft, and ventromedially inclined femoral head of many dromaeosaurids (*contra* [15]) (e.g., *Deinonychus* [176]; *Velociraptor* [106]). Even in unenlagiines and basal dromaeosaurids, such as *Buitreraptor*, *Microraptor* and *Rahonavis*, which have a straight femur in anterior view ([25], [109], [114]:fig. 27), the femoral head is inclined ventromedially, unlike in *T. hermani*. *T. hermani* also lacks fusion between the lesser and greater trochanters (a paravian synapomorphy, that is also present in some alvarezsaurids [87], [90] and oviraptorosaurs (J. N. Choiniere, pers. comm. January 2012)) and lacks an anteroposteriorly thick greater trochanter (a maniraptoran synapomorphy [87]). These observations preclude referral to Dromaeosauridae or any other maniraptoran clade. Thus, *T. hermani* can confidently be identified as a non-maniraptoran coelurosaur.

Rich & Vickers-Rich [41] referred *T. hermani* to Ornithomimosauria. However, NMV P186303 lacks several features present in all ornithomimosaurs, such as the ‘aliform’ lesser trochanter [15], [177] and prominent accessory trochanter [154], [162], [178]. In contrast, the lesser trochanter of some tyrannosauroids is anteroposteriorly narrower, and the accessory trochanter forms a transversely thickened region, similar to the condition in *T. hermani* (e.g., *Tyrannosaurus* [79]). *T. hermani* also possesses a proximomedially inclined (‘elevated’) femoral head, a synapomorphy of derived tyrannosauroids (e.g., [151]; *Tyrannosaurus* [79]; *Xiongguanlong* [161]), that is absent in ornithomimosaurs (e.g., [162], [178]). *T. hermani* resembles more basal tyrannosauroids [130], [156], as it lacks the deep extensor groove, a synapomorphy of the clade comprising *Xiongguanlong* and more derived tyrannosauroids [151], [161]. On this basis it can be placed within Tyrannosauroidea, but outside of a derived clade including *Xiongguanlong* and tyrannosaurids. This is similar to the phylogenetic position of the pubis NMV P186046 posited by Benson et al. [16], [20], and it is possible that both specimens represent a single taxon. Long, slender femoral proportions are not seen in other tyrannosauroids (e.g., [172], [175]). Thus, if *T. hermani* is a tyrannosauroid, its proportions may be autapomorphic. Even if it is not a tyrannosauroid, the combination of tyrannosauroid-like features and gracile proportions suggest that it should not currently be considered a *nomen dubium* (*contra* [15], [134]).

#### NMV P186323, Lake Copco Site, Dinosaur Cove

**Figure 20:**
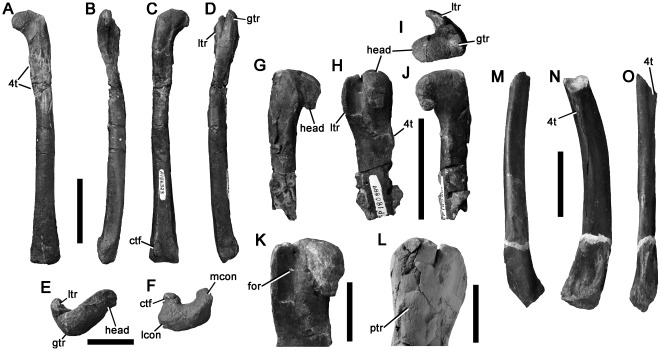
Theropod femora. Maniraptoran left femur NMV P186323 (A–F), proximal portion of an unenlagiine right femur NMV P180889 (G–L), and indeterminate coelurosaurian femur NMV P230845 (M–O) in anterior (A, G, M), medial (B, H, N), posterior (C, J), lateral (D, L, O), proximal (E, I), distal (F) and anteromedial (K) views Abbreviations: 4 t, fourth trochanter; ctf, crista tibiofibularis; for, foramen; gtr, greater trochanter; lcon, lateral condyle; ltr, lesser trochanter; mcon, medial condyle; ptr, posterior trochanter. Scale bars equal 50 mm (A–D, G–J, M–O) and 20 mm (E–F, K–L).

A small left femur was referred to *Timimus hermani* by Rich & Vickers-Rich [41] (Fig. 20A–F). The proximal half of the bone is crushed anteroposteriorly, but the distal portion is uncrushed. The distal and proximal ends, including the lesser trochanter, are abraded. Despite the much smaller size of NMV P186323, the proportions are similar to those of the holotype of *T. hermani* (NMV P186303), the ratio of femoral length to mediolateral width is 13.9. This does not preclude NMV P186323 from referral to *T. hermani* because gracile theropod femora scale isometrically [179]. The greater trochanter of NMV P186323 is anteroposteriorly broad relative to the lesser trochanter, suggesting that the anteroposterior width of the greater trochanter was at least subequal to that of the head, as in maniraptorans, but unlike the condition in non-maniraptoran theropods, including *T. hermani* (Fig. 19G) [87]. The anterior surface of the femoral head is abraded so this is difficult to confirm directly. Nonetheless, the proportional thickness of the greater trochanter of NMV P186323 is certainly greater than that of *T. hermani*, suggesting that the two are not conspecific. NMV P186323 also differs from *T. hermani* because the fourth trochanter is substantially weaker, reduced to a low ridge as in maniraptorans [87], and the extensor groove is completely absent. Although NMV P186323 is smaller than the holotype of *T. hermani*, and perhaps represents a juvenile, it is unlikely that these differences result from allometry or ontogeny. Ontogenetic series of the allosauroids *Fukuiraptor* [180] and *Allosaurus* (UMNH VP specimens listed by [158]), and of the tyrannosauroids *Tyrannosaurus* (T. Carr, pers. comm. 13 January 2012: the greater trochanter) do not show size-related changes in the thickness of the greater trochanter or prominence of the fourth trochanter. Unfortunately, however, there is currently little published data on coelurosaurian femoral ontogeny so this conclusion remains tentative. If the broad greater trochanter and low fourth trochanter of NMV P186323 are phylogenetically informative, then they indicate referral to Maniraptora, but the precise affinities of the specimen cannot be determined.

#### NMV P180889, Lungfish Site, Cape Lewis

The undeformed proximal portion of a right femur (Fig. 20G–L). The femoral head is inclined ventromedially and does not project as far medially as does that of *T. hermani* or many other theropods (e.g., [79], [88], [122]–[123]). The femoral head appears to be directed medially, although this cannot be determined with certainty because the distal femur is missing. The greater trochanter is substantially wider anteroposteriorly (15 mm) than the caput (12 mm), as in maniraptorans [87], and unlike in *T. hermani* (Fig. 19G). The posterior flange of the caput is abraded. The lesser trochanter is aliform, and its anterior surface is abraded, so the morphology of the accessory trochanter cannot be determined. As preserved, the lesser trochanter extends approximately to the level of the greater trochanter. However, the proximal portion of the lesser trochanter is broken, and it would originally have extended further, as in many coelurosaurs [87]. The lesser trochanter is partly ‘fused’ to the greater trochanter, as in paravians, some alvarezsaurids [87], [90], and some oviraptorosaurs (J. N. Choiniere, pers. comm. January 2012). These trochanters are only separated by a proximodistally short notch (measuring 9 mm laterally; less than one-third the proximodistal height of the trochanter; and 4 mm medially). A small foramen opens proximally on the anteromedially facing surface between the lesser trochanter and femoral head (Fig. 20). This is in the same location as the femoral pneumatic foramen of many birds (P. M. O'Connor, pers. comm. October 2011), but its small size suggests that it is a nutrient foramen. An identical foramen was listed as an autapomorphy of *Santanaraptor* by Kellner et al. [181]. However, the femur of *Santanaraptor* differs from P180889 in several important respects: the lesser trochanter does not extend as far proximally and the femoral head extends further medially [181]. A foramen in this location was also reported in the oviraptorosaur *Shixinggia*, in which it is proportionally larger and may have been pneumatic [182]. We suggest this foramen has a wide, but as yet undetermined, distribution among coelurosaurian dinosaurs.

A subtle, raised, subcircular, rugose eminence approximately 9 mm in diameter is located on the lateral surface of the femur posterior to the base of the lesser trochanter. This regarded here as homologous to the posterior trochanter of paravians [104], and it is only weakly developed in NMV P180889. The superficial bone layers proximal and posterior to the posterior trochanter are broken, accentuating its appearance. The preserved portion of the shaft is very thin-walled with a ‘hollow’ appearance. The fourth trochanter forms a low ridge, corresponding to the reduced morphology of maniraptorans [87], [97]. In some dromaeosaurids and parvicursorine alvarezsaurids the fourth trochanter is completely or almost completely absent [90], [109], [114], [168], [176], whereas in others it is weak (*Buitreraptor* [25]) or relatively prominent (*Velociraptor* [106]).

NMV P180889 differs from *Timimus* in its proximodistally shorter cleft between the greater and lesser trochanters, the anteroposteriorly broader, ‘aliform’ dimensions of the lesser trochanter, the presence of a foramen on the medial surface between the head and the lesser trochanter, the anteroposteriorly broad greater trochanter, the mediolaterally short, ventromedially inclined femoral head, and the low, ridge-like and proximally positioned fourth trochanter. The broad greater trochanter of NMV P180889 is a maniraptoran synapomorphy [87]. Extensive fusion of the greater and lesser trochanters is present in paravians, alvarezsaurids, and some oviraptorosaurs (in which the morphology of the lesser trochanter differs in being ‘cylindrical’ [121]) [87], [90], [112], and the posterior trochanter is a unique paravian synapomorphy [173] (although Novas & Puerta [168] reported its absence in *Unenlagia*, and it was not specifically described as present or absent in *Buitreraptor* [25] or *Rahonavis* [109]). Thus, NMV P180889 can confidently be referred to Paraves.

Among paravians, the femoral head is mediolaterally short, and inclined ventromedially in non-dromaeosaurine dromaeosaurids, including unenlagiines and microraptorans [25], [109], [114] (this also occurs in many non-tetanuran theropods [144], [173]). Although in dromaeosaurine dromaeosaurids, the femoral shaft curves proximomedially, in non-dromaeosaurines the shaft is straight [25], [109], [114], similar to the condition in NMV P180889. Based on these observations, NMV P180889 represents a non-dromaeosaurine dromaeosaurid, and is thus possibly referable to Unenlagiinae.

#### NMV P230845, 2 km east of Blanket Bay, near Cape Oway

A right femur missing the proximal end (Fig. 20M–O). The distal end is highly abraded. The fourth trochanter forms a low crest. The extensor groove is absent. The medial distal crest seems prominent, but may have been accentuated by crushing of the medial surface posterior to it. Because of the low fourth trochanter, and lack of otherwise diagnostic features, this specimen is referred to Coelurosauria indet.

### Tarsals

#### NMV P150070, 100 m west of Eagle's Nest

**Figure 21:**
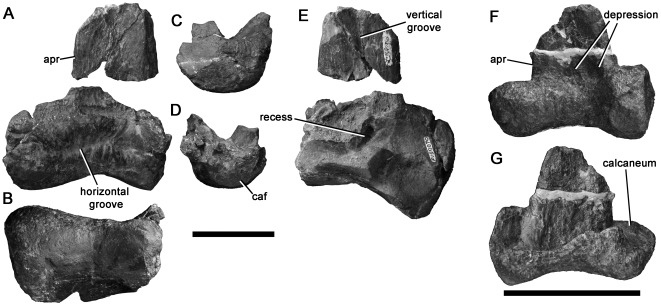
Theropod left proximal tarsals. Megaraptoran astragalus NMV P150070 (A–E) and ceratosaur astragalocalcaneum NMV P221202 (F–G) in anterior (A, F), ventral (B), medial (C), lateral (D), posterodorsomedial (E), and posterior (G) views. Abbreviations: apr, ascending process; caf, calcaneal facet. Scale bars equal 50 mm.

A left astragalus missing the basal portion of the ascending process. The posterodorsal portion of the body is broken and the calcaneal facet (lateral surface) is abraded posterodorsally (Fig. 21A–E). This specimen was described in detail by Molnar et al. [183] as an ‘allosaurid’. It was discussed extensively by Welles [184], Molnar et al. [185], and Agnolin et al. [15], and so is not described again here, except to summarise some anatomical points. Welles [184] suggested that NMV P150070 represents an ornithomimid and Agnolin et al. [15] suggested abelisauroid affinities. These arguments were refuted very clearly by Molnar et al. [185] and Fitzgerald et al. [186] and we emphatically support an assignment to Allosauroidea.

Megaraptoran allosauroids have only recently become well-known [21], [45], [89], [145]. Hocknull et al. [45] identified numerous features shared between NMV P150070 and the megaraptorans *Australovenator* and *Fukuiraptor*. Shared features include the proportionally tall ascending process and its dorsally tapering morphology, the presence and morphology of the horizontal groove on the anterior surface of the astragalar body, and the trapezoidal outline of the bone in distal view. These similarities suggest a close relationship between NMV P150070 and megraptorans [21], [45]. However, the astragali of more basal neovenatorids are not known, and the distal tibia of *Chilantaisaurus* suggests it possessed a similar astragalus to those of megaraptorans (the ascending process was likely of similar proportional height [21], [131]). Thus, we identify NMV P150070 as a neovenatorid allosauroid, but only possibly as a megaraptoran.

#### NMV P221202, San Remo

A small left astragalocalcaneum that is described in detail as a ceratosaur by Fitzgerald et al. [186] (Fig. 21F–G). We concur with this identification and the details provided in that work.

### Metatarsals

#### NMV P180856, Point Franklin, east of Cape Otway Lighthouse

**Figure 22:**
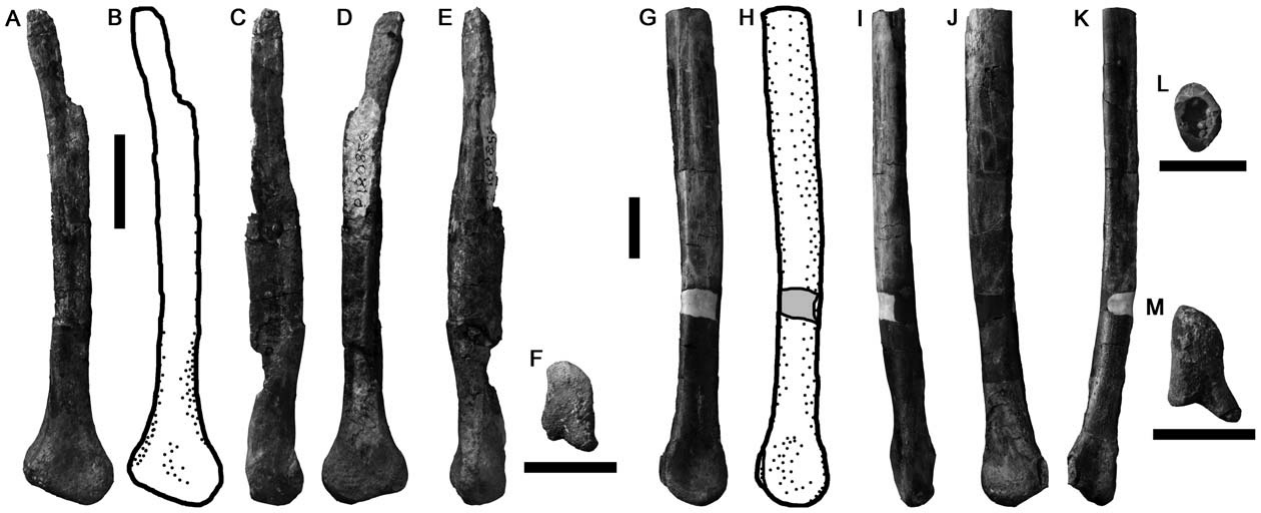
Metatarsals referred to Theropoda indet. NMV P180856 (A–F) and P209990 (G–M) in medial/lateral (A–B, D, G–H, J), ventral (C, K), dorsal (E, I), proximal sectional (L), and distal (F, M) views. Scale bars equal 20 mm.

The distal half of a gracile right metatarsal II, or left metatarsal IV (Fig. 22A–F). It is described here as metatarsal II, although it is difficult to confirm either identification. The proximal part of the specimen is eroded, so only the lateral surface is well-preserved, representing the mid-shaft region and distal portion of the bone. The mid-shaft region has a flat lateral surface that was appressed to metatarsal III. From here, the shaft curves distolaterally, and has a convex lateral surface that presumably did not contact metatarsal III. The cross-sectional dimensions are only apparent for this distal region (just distal to midlength of the specimen as preserved), in which the shaft is wider mediolaterally (12 mm) than dorsoventrally (8 mm) and has a trapezoidal cross-section, with a narrow dorsal surface and wide, flat ventral surface.

The distal condylus is transversely narrow and dorsoventrally tall, similar to the metatarsal IV of abelisauroids [143], [171] and various other theropods, including the allosauroid *Neovenator* [99]. It forms a convex articular surface that bifurcated ventrally into ventromedial and ventrolateral malleoli. Distinct ligament pits are absent but the lateral surface of the distal condylus is weakly concave. This specimen lacks diagnostic features of any particular clade and is considered as Theropoda indet.

#### NMV P209990, Flat Rocks Site, Inverloch

A gracile left metatarsal IV or right metatarsal II missing the proximal end (Fig. 22G–M). It is described here as metatarsal IV. The shaft is mediolaterally narrow proximally (ratio of mediolateral (11 mm) to anteroposterior (15 mm) diameter at one-third preserved length = 0.73) but becomes broader distally so that the mediolateral and anteroposterior diameters are subequal (∼12 mm at two-thirds preserved length). The ventral surface is approximately flat proximally and is inclined to face posteromedially. More distally, it is mediolaterally convex and forms a continuous curve with the lateral surface. The medial surface bears a proximodistally long concave facet for metatarsal III. This facet is dorsoventrally narrow proximally (4 mm), attains maximum breadth (9 mm) around midlength of the bone as preserved and terminates within a reconstructed area around two-thirds of preserved shaft length. The distal end of the metatarsal is anteroposteriorly tall and mediolaterally narrow. It forms a convex articular surface that is divided posteriorly into a robust medial malleolus and flange-like, posterolaterally oriented lateral malleolus. The outline in distal view is similar to the metatarsal IV of the abelisauroid *Masiakasaurus* [171] because of its mediolaterally narrow outline and strongly posterolaterally curving lateral ramus. However, the neovenatorid allosauroid, *Neovenator*, also possesses a similar morphology [99], which might be more widely distributed among theropods, so we refrain from drawing systematic conclusions here. The lateral surface of the distal end is gently depressed. The inner surface bears a distinct ligament pit. This specimen lacks diagnostic features of any particular clade and is considered as Theropoda indet.

### Pedal phalanges

#### NMV P186151, The Arch, near Kilcunda

**Figure 23:**
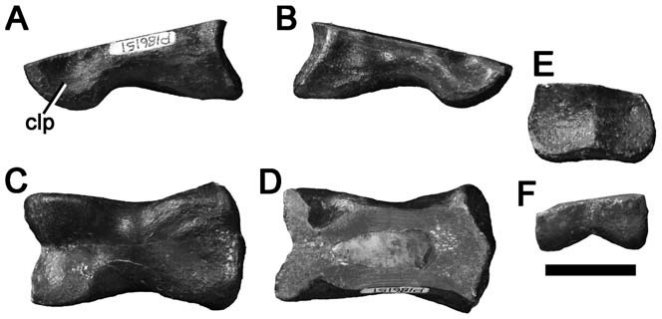
Pedal phalanx referred to Theropoda indet. NMV P186151 in left side (A), right side (B), ventral (C), dorsal (D), proximal (E), and distal (F) views. Abbreviation: clp, collateral ligament pit. Scale bar equals 20 mm.

A pedal phalanx that has been sectioned and polished so the dorsal part is missing (Fig. 23). It is transversely broad and dorsoventrally low. Deep collateral ligament pits are present. Because it lacks diagnostic features of any particular clade it is considered as Theropoda indet.

### Flat Rocks theropod teeth

**Figure 24:**
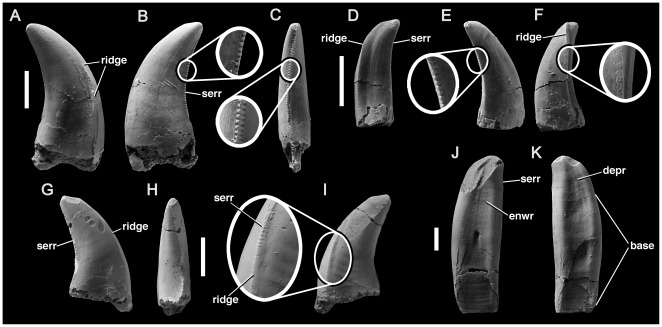
Theropod teeth from the Flat Rocks Site preliminarily referred to Megaraptora. NMV P186353 (A–C), P210084 (D–F), P212859 (G–I), and P230871 (J–K) in labial (A, G), lingual (B, D, I), distal (C, F, H), mesial (E) and undetermined (J–K) views. Scale bars equal 5 mm.

At least ninety theropod teeth have been collected from the Flat Rocks Site. Because their morphology suggests they originated from a single taxon that may be important for understanding Victorian theropod diversity, we briefly report this material here. All well-preserved teeth possess mesial and distal carinae (Fig. 24). The smallest tooth (NMV P221205) has a crown height of 3.5 mm, mesiodistal (‘fore-aft’) basal length of 2 mm and labiolingual width of 1 mm. The largest tooth (NMV P198947) has a crown height of 21 mm, mesiodistal basal length of 9.5 mm and labiolingual width of 5 mm. Two morphotypes are present, which respectively represent lateral (Fig. 24A–C, G–K), and premaxillary or other mesial (i.e. anterior) teeth (Fig. 24D–F). All well-preserved lateral teeth have mesial and distal carinae (N = 75, of which 42 preserve the full crown height; mean crown height = 11.1 mm). The distal carina is placed distolingually, extends to the base, and has a serration density that is higher in small teeth (NMV P221205 has 30 denticles/5 mm; extrapolated from 6 denticles/mm) than in large teeth (NMV P198947 has 16 denticles/5 mm). The mesial carina is unserrated, forming a narrow ridge, and curves labially on its course from apex to base. Fourteen premaxillary teeth are preserved, of which 10 preserve the full crown height (mean crown height = 10 mm; suggesting they are ∼90% the height of lateral teeth). Like the lateral teeth, premaxillary teeth possess unserrated mesial and serrated distal carinae. Both are located lingually, giving the tooth a ‘D-shaped’ cross section. Most teeth lack enamel wrinkles (as defined in [187]). However, weak, band-like wrinkles are present in at least nine lateral teeth (Fig. 24G–K) (NMV P186457, P198947, P198958, P199070, P210025, P212859, P221204, P229111, P230871). This likely represents intraspecific variation, as observed in *Megalosaurus* [158]. Futher evidence for intraspecific variation is given by the presence of weak serrations on some portions of the mesial carina of NMV P212859 (Fig. 24I). These serrations have a similar density to those of the distal carina. One tooth preserves most of the base (NMV P230871). As in other theropods, the labial and lingual surfaces of the base are depressed centrally (Fig. 24J–K), yielding a ‘figure of eight’ cross section. These depressions extend a short distance apically onto the crown in some of the teeth.

The ‘D-shaped’ cross-section of premaxillary teeth from Flat Rocks is similar to some allosauroids, including *Australovenator*, *Fukuiraptor* and *Orkoraptor* [45], [133], [180], and some coelurosaurs, including tyrannosauroids [84], [175]. The absence of enamel wrinkles on most teeth from Flat Rocks differs from many tetanurans [187], but is similar to *Australovenator* [45]. The Flat Rocks Site teeth differ from the only well-known Australian theropod, the megaraptoran *Australovenator*, in possessing an unserrated, or only partially serrated (Fig. 24I) mesial carina (the serrated mesial carina is most clearly shown in *Australovenator* [45]:fig. 20E). Although high density, small-sized denticles, which might be described as ‘reduced’, have been reported in dromaeosaurids and some basal tyrannosauroids [188]–[189], this differs from the absence of serrations on the mesial carina of most Flat Rocks Site teeth. Denticulation of the mesial carinae of the Flat Rocks site teeth differs still further from that in dromaeosaurids, because rare examples are weakly serrated, and their denticles occur at sizes and densities comparable to those of the distal carina (Fig. 24I). Identification of the Flat Rocks site teeth, and of teeth from other sites, requires more detailed comparative work that is being undertaken by S. W. Salisbury, P. J. Currie, and F. E. Novas.

## Discussion

### Taxonomic composition and diversity of a high latitude Gondwanan theropod fauna

Several previous works have described and identified theropod material from the Early Cretaceous of Victoria. Together, these report high clade diversity, including Ornithomimosauria [41], Oviraptorosauria [43], Megaraptora [19], [21], [45], [186], Dromaeosauridae and Ceratosauria [15], [186], Tyrannosauroidea [16], and Spinosauridae [17]. However, these studies do not present a unified consensus (e.g., [15], [21]–[22], [183]–[186]). This has arisen from the absence of an overarching review, and paucity of detail in many published figures and descriptions, that has led to differences in interpretation (for example, we have clarified several aspect of the anatomical descriptions of Agnolin et al. [15]). In the present work, we attempted to provide detailed figures, and synapomorphy-based identifications, to help resolve these disputes.

We identify up to seven theropod clades in the Victorian assemblage: Ceratosauria (NMV P221202), Spinosauridae (NMV P221081), Tyrannosauroidea (NMV P186046, P186303), Maniraptora, including Paraves (Tables 1–2; several specimens), a basal coelurosaur (NMV P180880), possibly Ornithomimosauria (NMV P186168), and Allosauroidea (Tables 1–2; several specimens, which are referred to Neovenatoridae or the neovenatorid subclade Megaraptora, and may represent a single taxon or multiple taxa). Because dorsal vertebrae represent at least two non-dromaeosaurid maniraptorans (NMV P186302, P216672), and NMV P180889 is likely a dromaeosaurid femur, a minimum of nine theropod taxa appear to be present. The most conservative estimate of taxonomic diversity is based on the presence of four morphologically distinct dorsal vertebrae (Figs. 4, 5, 6, 7), suggesting that at least four taxa are present. This suggests high taxonomic diversity of theropods at high paleolatitudes represented by the Victorian deposits (75–80 degrees South [62]–[63]).

Remains of large-bodied theropods include a manual ungual phalanx (Fig. 15; NMV P186153) comparable in size to that of an 8.5 metre long individual of *Baryonyx* [85], a coracoid (Fig. 12; NMV P186327), an astragalus (Fig. 21A–E; NMV P150070), several distal caudal vertebrae (Fig. 11; Table 1), elongate manual phalanges (Fig. 14; Table 2), and a ‘medium-sized’ but ontogenetically immature dorsal centrum (Fig. 4; NMV P221187). Most of this material can be referred to either Avetheropoda indet. (the coracoid), or Neovenatoridae indet. (the dorsal vertebra, astragalus and ungual phalanx), and one element distinctly differs from those of megaraptorans (the ungual phalanx). This suggests the presence of a large-bodied basal neovenatorid (Tables 1–2). An ulna (Fig. 13; NMV P186076) represents a smaller-bodied individual, and is strikingly similar to those of megaraptoran neovenatorids [19], [45]. It is possible that this ulna represents a megaraptoran, and if this is correct then it is possible that two allosauroids are present in the Victorian assemblage. A pneumatic middle caudal vertebra (NMV P208096; Fig. 10) may also represent a megaraptoran or more basal neovenatorid. The abundance of allosauroid remains, including specimens from Flat Rocks, raises the possibility that abundant teeth from the Flat Rocks locality may also belong to allosauroids. The largest tooth has a crown height of 21 mm and fore-aft basal length of 10 mm, considerably smaller than the teeth of giant theropods. Thus, the Flat Rocks teeth suggest a medium-sized megaraptoran neovenatorid, consistent with the size of the ulna NMV P186076 and supporting the hypothesis of multiple allosauroid taxa. However, we cannot provide a decisive count of allosauroid taxa based on present evidence.

Other large-bodied theropods were also likely present, as indicated by a juvenile spinosaurid cervical vertebra (Fig. 3; NMV P221081 [17]; all known spinosaurids achieved large adult body sizes). This spinosaurid, and other taxa, are represented only by single specimens. For example, specimens referred to Ceratosauria (Fig. 21F–G; NMV P221202 [186]), an ornithomimosaur-like caudal vertebra (Fig. 9; NMV P186168), and maniraptoran remains representing at least two, and possibly three distinct taxa (Figs. 5–6, 20; NMV P186302, P180889, P216672). These are primarily small- or medium-sized individuals. Abundant singleton representation (the presence of several taxonomic groups represented only by a single specimen) contrasts with the situation for large-bodied theropods and suggests highly incomplete faunal sampling of small-bodied taxa. Thus, the Victorian fauna is characterised by high theropod diversity at small body size, and future discoveries can be expected to yield still higher taxonomic diversity.

### Understanding Early Cretaceous biogeography

Bonaparte [23]–[24] proposed that during the Cretaceous, Gondwanan and Laurasian tetrapod faunas were distinct at high taxomic levels. Vicariance played a central role in his biogeographic model, although the taxonomic level considered as important varied among different groups. For example, although many clades were proposed as Gondwanan ‘endemics’, he also posited that some clades had initially Pangean distributions, but contained endemic genera. This model is now widely-accepted. The ‘characteristic’ family-level clades of each continental bloc are usually considered to have arisen locally, following the formation of oceanic barriers between Laurasia and Gondwana.

However, since Bonaparte's work, new discoveries and phylogenetic reappraisals have expanded the distributions of many dinosaurian clades previously thought to be restricted to either Laurasia or Gondwana. For example, among theropods, ‘Gondwanan’ abelisauroids are present in both Early and Late Cretaceous Europe [190]–[191], and carcharodontosaurid allosauroids are now known in Asia and North America [21], [26]. Among sauropods, ‘Gondwanan’ titanosaurians are abundant and taxonomically diverse in Laurasia (e.g., [192]). Of course, northern and southern faunas were not identical, but as sampling of the dinosaur fossil record has improved, increasing numbers of taxonomic groups have been discovered in both hemispheres. Thus, many clades found in Cretaceous deposits attained a cosmopolitan distribution in the Jurassic, prior to continental fragmentation. Because these groups represent deep phylogenetic divergences that predate continental fragmentation, a simple assessment of their presence or absence cannot inform the inference of continent-scale vicariance [16]–[17], [27]. Indeed, such an approach, as employed recently by Agnolin et al. [15], is highly problematic, because the absence of some of these groups may arise from local extinction (as opposed to local origination, implicit in hypotheses of vicariant evolution) or intracontinental environmental preferences (e.g., [34]). Furthermore, this approach may be biased by our relatively incomplete knowledge of Gondwanan Cretaceous ecosystems compared to those of Laurasia (a similar observation has been made for Mesozoic mammals [193]). This is especially true for groups whose representatives are ecologically rare, or taphonomically fragile due to small body size (e.g., most coelurosaurs). Accurate biogeographic patterns must be elucidated by cladistic biogeographic methods, drawing on more recent, shallow phylogenetic divergences (e.g., [7]–[8]).

Unfortunately, few Australian Cretaceous vertebrates are sufficiently well-known to be included in phylogenetic analyses, and the low-level relationships of most taxa are disputed, or do not provide decisive evidence for affinities with any particular global region. For instance, among archosaurs, the crocodyliform *Isisfordia* is the sister taxon of all other eusuchians, which comprise both Laurasian and Gondwana taxa [194]–[195]. *Australovenator* [45], a megaraptoran theropod, may be the sister taxon of *Fukuiraptor*, from Japan. And the sauropods *Wintonotitan* and *Diamantinasaurus* may be related to Laurasian or Gondwanan taxa [45]. Until there is consensus on these phylogenetic issues, and until the Australian Cretaceous fauna is more completely known, it will be difficult to make any convincing statements about vicariance and the Australian Cretaceous tetrapod fauna.

### Comparison of the Victorian Early Cretaceous theropod fauna

Our appraisal of the Victorian theropod fauna indicates abundant neovenatorid allosauroids, possibly representing two taxa: a large-bodied non-megaraptoran and a medium-sized megaraptoran. Also present were rare ceratosaurs, possibly spinosaurids, and diverse small–medium sized coelurosaurs, including tyrannosauroids and a possible ornithomimosaur, two clades currently unknown from other Gondwanan continents (although the Brazilian *Santanaraptor* may be a tyrannosauroid [175]). The Gondwanan coelurosaur fauna includes small, basal coelurosaurs from Brazil, alvarezsaurids and unenlagiine dromaeosaurids from Patagonia (reviewed by Novas [14]), and isolated remains of Australian maniraptorans reported here. Unfortunately, most Victorian coelurosaur specimens are insufficiently diagnostic to determine affinities with Gondwanan or Laurasian forms, although the partial femur NMV 180889 may represent an unenlagiine.

The presence of megaraptoran allosauroids in the Victorian theropod fauna is similar to the faunas of Queensland and Patagonia. The only known theropod from Queensland, *Australovenator*, is a megaraptoran [21], [45], and several megaraptoran specimens are known from the Late Cretaceous of Patagonia [89], [133], [136], [146] (fewer fossils are known from Early Cretaceous Patagonian rocks). However, striking differences may also exist. Non-megaraptoran neovenatorids, possible large-bodied predators of Victoria, are otherwise only known from Laurasia [21] (although we also allow the possibility that these large-bodied Victorian theropod remains also pertain to megaraptorans). In the Early–early Late Cretaceous of South America and Africa, this ecological role was instead filled by carcharodontosaurine allosauroids (e.g., [39], [96], [196]–[197]. Carcharodontosaurines possess highly diagnostic teeth, which are relatively common fossils in North Africa and South America, but are not recorded in Victoria, or elsewhere in Australia (e.g., [37], [198]–[199]).

The most conspicuous difference between the theropod fauna of Victoria, and those of all other Gondwanan continents, is the absence or rarity of abelisauroid ceratosaurs [14], [23], [200]–[202]. Because abelisauroids diverged from tetanurans early in theropod evolution, they have highly characteristic anatomy that can be often recognised from isolated teeth (e.g., [171], [203]) and phalanges [200]. Thus, their scarcity in the Victorian fauna suggests genuine absence or rarity. Although the astragalus NMV P221202 represents a ceratosaur, it cannot be referred definitely to Abelisauroidea [186].

Appraisal of the dinosaur fauna as a whole yields a further conspicuous distinction; Victoria yields a high abundance and diversity of small-bodied ornithischians [60], [204], which differs from the sauropod-dominated faunas of most other regions of Cretaceous Gondwana [14], [23], [205], but is similar to Laurasian Cretaceous faunas, and that of terminal Cretaceous Antarctica (Campanian–Maastricthian [206]). In fact, sauropods are completely absent from the Victorian dinosaur fauna, despite the recovery of over 1500 dinosaur specimens. This is unusual, not just compared with other Gondwanan faunas, but also compared to the penecontemporaneous (latest Albian) fauna of the Winton Formation of Queensland, Australia, which has yielded multiple sauropod taxa. It is possible that the absence of Victorian sauropods reflects taphonomic bias against the preservation of large specimens. However, evidence for large-bodied theropods (NMV P186153, P150070), and the absence of sauropod teeth and other small elements (e.g., the bones of juveniles, cranial bones) both suggest genuine ecological absence.

### Biogeographic implications of the Victorian theropod fauna: climatic influence on Early Cretaceous dinosaur biogeography

The classic ‘Gondwanan’ fauna described by Bonaparte [23] was based primarily on observations of the Cretaceous tetrapod faunas of South America and Africa. These observations were extended to the Late Jurassic theropod fauna of Tanzania by Rauhut [207] and partly in [208]. During the Early Cretaceous, much of Africa and South America, including Patagonia, were within a large, arid climatic belt, which extended to low latitudes of North America and Asia ([209]:250–254). Queensland experienced a more humid, warm mid-latitude climate, and Victoria was located at polar latitudes, which experienced a temperate to frigid climate. Geological evidence suggests that, like Australia, much of Early Cretaceous Laurasia was characterized by a non-arid, warm humid or cool temperate climate ([209]:250–254). Thus, a climatic gradient may explain differences in taxonomic composition between the faunas of Victoria and other Gondwanan regions. Under this model, some clades classically considered as ‘Laurasian’, which are not yet known from South America or Africa, but are found in the Victorian fauna (e.g., Tyrannosauroidea), may have preferred higher, temperate and polar latitudes, or generally more humid biomes, in both hemispheres. The Early Cretaceous representatives of Abelisauroidea may have exhibited a strong environmental preference for arid biomes, and Sauropoda may have been very rare at high polar latitudes during the Cretaceous (although a single sauropod vertebra is now known from the Late Cretaceous of Antarctica [210], when global and polar temperatures were substantially higher [209]). Mannion et al. [35] demonstrated that sauropods were substantially less diverse at high latitudes in the Late Cretaceous, and it is likely that this was also true earlier in their history. Further evidence that clade-representation in Cretaceous dinosaur faunas was influenced by climatic zonation comes from the works of Mendeiros et al. [211] and Novas et al. [14], [212], who observed common faunal elements of ‘middle’ Cretaceous Brazil and North Africa, not represented in Patagonia. These include the presence of spinosaurids, *Ouranosaurus*-like ornithopods, and dyrosaurid crocodyliforms. They explained these differences as resulting from latitudinally varying factors such as temperature or humidity [14], [211]–[212].

Climatic controls on the distribution of higher-taxa have also been documented in Cretaceous Crocodyliformes, Mammalia, and plants. Notosuchian crocodyliforms are often considered as a ‘Gondwanan’ clade [15], [23], but Carvalho et al. [213] demonstrated that within Gondwana they were restricted to the arid climatic zones. Thus, they are absent from the Australian fauna both in Victoria and Queensland (only a stem eusuchian, *Isisfordia*, is known [194]). Rich et al. [214] observed endemicity at high taxonomic levels among Early Cretaceous (Aptian–Albian) Australian mammals. Of five families represented, only one is known outside Australia. They noted that similar regional effects are also apparent in Aptian–Albian paleo floras, for which the Australian and Antarctic records are compositionally similar, but are both markedly different to those of South America [215]–[217]. Nothing useful can be inferred about Late Cretaceous Australian dinosaurs, but mammals [214] and the palynological record of plants [218] show interchange between Australia (plus Antarctica) and South America in the Late Cretaceous, which may have arisen during a cool phase following the Turonian thermal maximum [214].

If the distribution of Early Cretaceous climates strongly influenced dinosaur distributions, this could introduce a misleading bias into those biogeographic studies that attribute all heterogeneity in clade distibutions to vicariance. This will also strongly bias studies reliant on phenetic comparisons of the presence or absence of particular clades (e.g., [15], [23]). We are not suggesting that continental fragmentation did not influence Cretaceous tetrapod biogeography, but that it only provides a partial explanation, in which the role climatic barriers has been underemphasised. In fact, if a high proportion of Gondwanan land area was intially characterised by arid biome, and Laurasia by more humid biomes, then the interaction of global climate distribution and oceanic barriers formed by continental fragmentation may have acted together to determine the overall character of Cretaceous faunas of the northern and southern hemispheres.

We support the suggestion that many higher clades of theropod dinosaurs (e.g., Carcharodontosauria, Spinosauridae, Abelisauroidea, Tyrannosauroidea) attained a global or near-global distribution in the Late Jurassic, and thus do not provide useful data for appraising the existence of vicariance in dinosaur distributions [17], [26]–[27], [32], [33]. Instead, the relationships among lower-level taxa, representing Cretaceous evolutionary divergences, should be used to support hypotheses of vicariance. For this to be effective, greater sample sizes may be required for many clades. Regional representation, and the relative abundances and diversities of some clades might have been controlled by climatic zonation. We suggest that some clades classically considered as ‘Gondwanan’, including Abelisauroidea, were actually most abundant and diverse at low, arid latitudes, which characterize most of Early Cretaceous Africa and South America, but were less widespread in Laurasia, and did not extend as far south as Australia.
